# Indole and azaindole halogenation catalyzed by the RebH enzyme variant 3-LSR utilizing co-purified *E. coli* reductase

**DOI:** 10.3389/fbioe.2022.1032707

**Published:** 2022-12-01

**Authors:** Eunice Hui Yen Li, Barindra Sana, Timothy Ho, Ding Ke, Farid J. Ghadessy, Hung A. Duong, Jayasree Seayad

**Affiliations:** ^1^ Institute of Sustainability for Chemicals, Energy and Environment, A*STAR, Singapore, Singapore; ^2^ Disease Intervention Technology Laboratory, Institute of Molecular and Cellular Biology, A*STAR, Singapore, Singapore

**Keywords:** halogenase, indole, azaindole, halogenation, bromination, enzyme, co-purified reductase

## Abstract

Biocatalytic C-H halogenation is becoming increasingly attractive due to excellent catalyst-controlled selectivity and environmentally benign reaction conditions. Significant efforts have been made on enzymatic halogenation of industrial arenes in a cost-effective manner. Here we report an unprecedented enzymatic halogenation of a panel of industrially important indole, azaindole and anthranilamide derivatives using a thermostable RebH variant without addition of any external flavin reductase enzyme. The reactions were catalyzed by the RebH variant 3-LSR enzyme with the help of a co-purified *E. coli* reductase identified as alkyl hydroperoxide reductase F (AhpF).

## Introduction

Halogenated arenes serve as versatile building blocks in organic synthesis and key structural motifs in numerous natural products and drug molecules (Butler and Walker, 1993; Stürmer, 1999; Littke and Fu, 2002; Nicolaou et al., 2005). A large proportion of drugs in clinical trials or on the market at present contain halogen atoms, which are known to have a profound effect on the bioactivity and physicochemical properties of small molecules (Lu et al., 2012; Xu et al., 2014). Efficient methods for selective installation of halogen substituents are thus highly desirable. Traditional halogenation methods often require electrophilic halogen sources that are hazardous and/or harmful to the environment. Stereo and regioselectivity is another challenge in many cases whereby a mixture of isomers is obtained. Enzymatic halogenation offers a highly attractive alternative, allowing green chemistry principles (van Pée, 2012; Schnepel and Sewald, 2017; Latham et al., 2018; Fejzagić et al., 2019; Crowe et al., 2021; Paul et al., 2021) such as less hazardous chemical synthesis and use of safer solvents. For example, the use of halogenases in aqueous reaction media at ambient temperatures minimizes the use of toxic volatile organic solvents. Moreover, the reaction employs benign halide salts as the halide source avoiding hazardous halogenation reagents and exhibits excellent regioselectivity thereby simplifying the product purification process.

**FIGURE 1:**
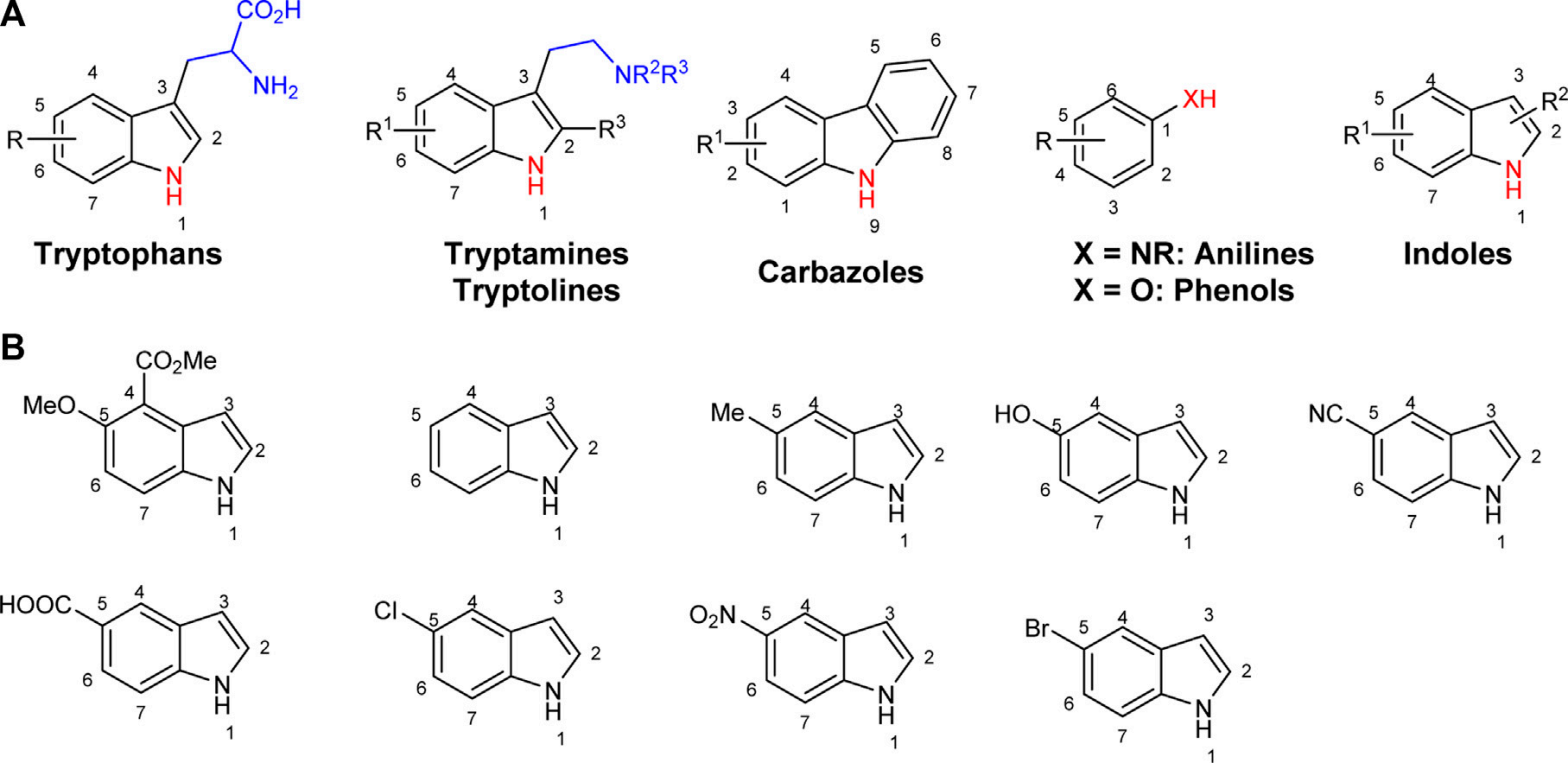
Arenes previously reported in halogenase studies; **(A)** Classes of compounds accepted by halogenases, **(B)** Indoles without a side chain at C2 and C3 as substrates for halogenases.

To explore the synthetic utilities of halogenases, significant efforts have been directed to investigate the substrate scope of these enzymes, and several classes of electron rich arenes such as tryptophans, tryptamines, tryptolines, anilines, phenols and indoles have been shown to serve as substrates for halogenases (Figure 1A) (Hölzer et al., 2001; Payne et al., 2013; Payne et al., 2015; Shepherd et al., 2015; Andorfer et al., 2016; Andorfer et al., 2017; Fisher et al., 2019; Gkotsi et al., 2019). For anilines and phenols, halogenation generally occurs at the most electrophilic sites (*o-* and *p*-substituted). When there is a strong interaction between halogenase and the side chain of substrate at the C3 position (i.e., tryptophans, tryptamines and tryptolines), halogenation can be directed at less electrophilic positions, for example C5, C6 or C7 (Dong et al., 2005; Lang et al., 2011; Payne et al., 2015; Andorfer et al., 2016). The halogenation of indoles without a side chain at C2 and C3 positions is not well-studied, and most examples are limited to chlorination. Lewis et al. used two halogenase enzymes RebH and Thal for chlorinating several indole derivatives such as indole and 5-methylindole (Figure 1B) (Payne et al., 2013; Payne et al., 2015; Andorfer et al., 2017). Sewald and co-workers identified BrvH, a new FDH, for the C3-halogenation of free indole, which showed significantly higher bromination activity over chlorination (Neubauer et al., 2018). For instance, full conversion of indole to bromoindole was observed in 48 h while the chlorination proceeded only to 8.4% under similar conditions. Recently, they also extended the substrate scope of BrvH to other indole derivatives *via* virtual screening (Figure 1B) (Neubauer et al., 2018; Neubauer et al., 2020). The same group also identified Xcc halogenases from *Xanthomonas campestris* pv. Campestris B100 could brominate 5-hydroxyindole, 5-methylindole and indole-5-carbonitrile at C3-position with moderate to low activity (Ismail et al., 2019). We report herein the halogenation of an extended panel of indole, azaindole and anthranilamide derivatives with RebH 3-LSR (3-LSR hereafter), a thermostable variant of RebH, with focus on production of brominated compounds, which are highly valuable precursors for transition metal-catalyzed cross-coupling reactions (Miyaura and Suzuki, 1995; Torborg and Beller, 2009; Poor et al., 2014). Additionally, the halogenation reactions were carried out by using endogenous *E. coli* reductase, identified as AhpF, that co-purified with the recombinant 3-LSR enzyme, thereby simplifying the enzyme catalyzed process further by eliminating the need for any externally produced and purified Fre enzyme.

Flavin-dependent halogenases (FDHs), which require only a halide salt, molecular oxygen and FADH_2_ as a cofactor, have been emerging as a highly promising synthetic tool (van Pée and Patallo, 2006). The expensive cofactor FADH_2_ can be regenerated *in situ via* the use of NADH-dependent flavin reductase (Fre). This cofactor regeneration is essential to maximize the product formation using minimum quantities of FADH_2_, which typically needs the recombinant production and purification of the Fre enzyme. Regeneration of NADH can be further achieved with an appropriate enzyme such as glucose dehydrogenase (GDH) using glucose as the terminal reductant. The native RebH partner RebF is widely used for FADH_2_ regeneration in RebH-catalyzed halogenation reactions. Although functionally linked and derived from same biosynthetic gene cluster, RebF does not have any direct interaction with RebH. The role of RebF is simply to supply reduced FADH_2_ to RebH, and could potentially be replaced by alternative Fre enzymes. Purified flavin reductases from various microorganisms including *E. coli* were reported to be used for cofactor regeneration in enzymatic halogenation reactions (Zeng and Zhan, 2010; Menon et al., 2016; Menon et al., 2017). In another approach, crude or partially purified recombinant halogenases were successfully tested for catalyzing halogenation reactions without addition of any external Fre, possibly by utilizing an endogenous reductase from *E. coli* (Keller et al., 2000; Domergue et al., 2019). Although a particular well-studied *E. coli* Fre is widely used for the former application (Spyrou et al., 1991), various reductases could potentially be involved when crude or partially purified halogenases are used. It is worth mentioning that multiple Fres are generally present in every organism and thus in the crude recombinant enzyme preparations (van Pée, 2012).

## Methods

### General Information

Chemicals and anhydrous solvents were obtained from Sigma Aldrich and were used without further purification. Spectroscopic grade solvents were purchased from Sigma Aldrich. LC–MS analysis was performed by using an Agilent 6120 mass spectrometer with an Agilent 1290 quaternary gradient system. GC–MS analysis was performed by using an Agilent HP-7890 instrument with an Agilent HP-5975 with triple-axis detector and HP-5MS capillary column by using helium as the carrier gas. High-resolution mass spectra (HRMS) were recorded on an Agilent ESI-TOF mass spectrometer at 3500 V emitter voltage. Exact m/z values are reported in Daltons. Crude product and HPLC purified fractions were freeze dried using a Labconco lyophiliser at -84°C and 0.01 mbar vacuum.

### 3-LSR Expression

The 3-LSR gene was codon optimized for expression in *E. coli* (sequence is presented in Supplementary Table S1) and cloned in kanamycin-resistant pET22b plasmid in between NdeI and XhoI restriction sites, and 6-His tag was incorporated at the C-terminus. *E. coli* BL21 (DE3) competent cell was transformed with the plasmid construct, and used for 3-LSR protein production. For overexpression, LB broth containing 50 μg ml^−1^ kanamycin was inoculated with 1% (v/v) overnight culture and incubated at 37°C with constant shaking at 180 rpm. The culture was induced with 0.5 mM IPTG when Abs_600_ reached to 0.5, and over-expressed overnight at 25°C. The cells were harvested by centrifugation and stored at –80°C.

### HisTrap affinity chromatography

The cells were re-suspended in HisTrap buffer A (50 mM Tris-Cl + 500 mM NaCl +20 mM imidazole, pH 7.4) and lysed by sonication. Cell debris and insoluble materials were removed by centrifugation at 20,000 g for 30 min. The proteins were purified using His GraviTrap column (GE healthcare) following manufacturer’s protocol, and the protein was eluted in buffer B (50 mM Tris-Cl + 500 mM NaCl +500 mM imidazole, pH 7.4). Purity of eluted fractions was analyzed by SDS-PAGE. The fractions containing 3-LSR protein were pooled together and concentrated using 10 kDa MWCO Amicon Ultra centrifugal device and buffer exchanged to 50 mM phosphate buffer pH 7.5. Concentration of the purified protein was determined from Abs_280_.

### Ion-exchange chromatography

The ion-exchange chromatography was done using RESOURCE Q column (GE Healthcare) in AKTA purifier system. The HisTrap purified protein was buffer exchanged and diluted 10x in buffer A (25 mM Tris-Cl buffer, pH 8.6), and used as sample for binding in the RESOURCE Q column pre-equilibrated with the same buffer. Unbound proteins were removed by washing with 10 column volume buffer A and the bound proteins were eluted by linear gradient using increasing concentration of buffer B (buffer A+ 1M NaCl) and eluent was collected in 1 ml fractions. The fractions were analyzed by SDS-PAGE; the fractions containing 3-LSR proteins were pooled together and concentrated prior to further purification by size exclusion chromatography.

### Size-exclusion chromatography

The purification was done with HiLoad 16/600 Superdex 75 pg column in AKTA purifier system, using 20 mM phosphate buffer (pH 7.2) containing 200 mM NaCl as mobile phase. 2 ml concentrated eluent from RESOURCE Q column was injected to the pre-equilibrated column and eluted using the same buffer. 1 ml fractions were collected and analyzed by SDS-PAGE.

### Mass spectrometry

The C7 fraction from ion-exchange purification was buffer exchanged to 50 mM ammonium bicarbonate buffer, trypsin digested and peptides were extracted according to standard techniques (Bringans et al., 2008). Peptides were analysed by electrospray ionisation mass spectrometry using the Shimadzu Prominence nano HPLC system [Shimadzu] coupled to a 5,600 TripleTOF mass spectrometer [Sciex]. Tryptic peptides were loaded onto an Agilent Zorbax 300SB-C18 column (3.5 μm) and separated with a linear gradient of water/acetonitrile containing 0.1% HCOOH. Spectra were analysed to identify *E. coli* proteins using Mascot sequence matching software (Matrix Science) with UniProt database. The mass spectrometry proteomics data have been deposited to the ProteomeXchange Consortium using the MassIVE (Wang et al., 2018) partner repository, with the accession number doi:10.25345/C51G0J027.

### NMR spectroscopy

Proton (^1^H NMR, 400 MHz), carbon (^13^C NMR, 100 MHz) magnetic resonance spectra were recorded on Bruker Avance III 400 MHz spectrometer using CD_3_OD and (CD_3_)_2_SO. Chemical shifts for protons are reported in parts per million and are referenced to residual proton in solvent (^1^H NMR: CD_3_OD at 3.31 ppm and (CD_3_)_2_SO at 2.50 ppm). Chemical shifts for carbons are reported in parts per million and are referenced to the carbon resonances of the residual solvent peak (^13^C NMR: CD_3_OD at 49.00 ppm and (CD_3_)_2_SO at 39.52 ppm). Data are reported in the following order: chemical shifts are given (δ); multiplicities are indicated as s (singlet), d (doublet), t (triplet), q (quartet) and m (multiplet).

### RebF expression and purification

The RebF gene with C-terminus 6-His tag was cloned in pET22b plasmid, and transformed in *E. coli* BL21 (DE3). Single colony of transformant was used to make 10 mL overnight cuture in LB medium containing 50 μg/mL kanamycin. The overnight culture was diluted 100× into 1,000 mL of the same media, and was grown at 37°C with constant shaking at 180 rpm until Abs_600_ reaches to 0.5. The induction was performed with 0.1 mM IPTG and the culture was incubated for overnight at 25°C with shaking at 180 rpm. Cells were then harvested by centrifugation and stored at –20°C prior to purification. The protein was purified by HisTrap affinity chromatography as described above.

### AhpF expression and purification

The gene was codon optimized for expression in *E. coli*, and cloned by In-Fusion cloning in pET22b plasmid double digested by NdeI and XhoI. The protein was over-expressed in *E. coli* BL21 (DE3) at 16°C overnight using 0.5 mM IPTG, using LB-amp media. The protein was purified to homogeneity in three steps: HisTrap affinity chromatography, anion exchange chromatography and size exclusion chromatography, similar to 3-LSR purification.

### Analytical LC-MS Method

Crude mixture (20 μl) was injected onto C18 analytical column (1.8 *μ* packing, 2.1 × 50 mm, Agilent EclipsePlus). Gradient conditions of 10% MeCN/H_2_O (plus 0.1% HCOOH) held for 1 min followed by development to 95% MeCN/H_2_O over 3 min and reequilibration to starting conditions over 2 min. Column temperature and flow rates were kept constant at 25°C and 0.4 mL min^−1^ respectively. UV absorbance was detected at 254 nm and 280 nm throughout.

### Semi-preparative HPLC method

900 μl of solution containing crude mixture dissolved in MeOH was injected onto a semi-preparative C12 HPLC column (4 μ packing, 250 × 10 mm, Phenomenex Jupiter). Starting conditions of 40% MeCN/H_2_O (plus 0.1% TFA) were held for 2 min Before development to 90% MeCN/H_2_O over 20 min 95% MeCN/H_2_O then held for 3 min Prior to re-equilibration of starting conditions over 3 min. Flow rates were kept constant at 5 mL min^−1^. UV absorbance was detected at 220 nm throughout.

### Analytical scale biotransformation

2.0 mM substrate was mixed with NaBr (100 mM), glucose (20 mM), FAD (300 µM), 3-LSR enzyme (50 µM), Fre (5 µM), NADH (100 µM) and glucose dehydrogenase enzyme (0.7 µM) in 20 mM phosphate buffer to a total volume of 200 µl. The mixture was incubated overnight at 25°C and shaked at 180 rpm. The reaction was stopped by heating at 95 °C for 10 min and the precipitates were removed by centrifugation at 13,000 rpm for 10 min. Methanol (200 µl) was added to the supernatant and analysis was performed using analytical LC-MS method.

### Preparative scale biotransformation

The above reaction was scaled-up to 4 ml total volume and the mixture was lyophilized after removing the protein by heat treatment and centrifugation. The resultant supernatant was then mixed with methanol and purified by semi-preparative HPLC method.

## Results and discussion

**FIGURE 2:**
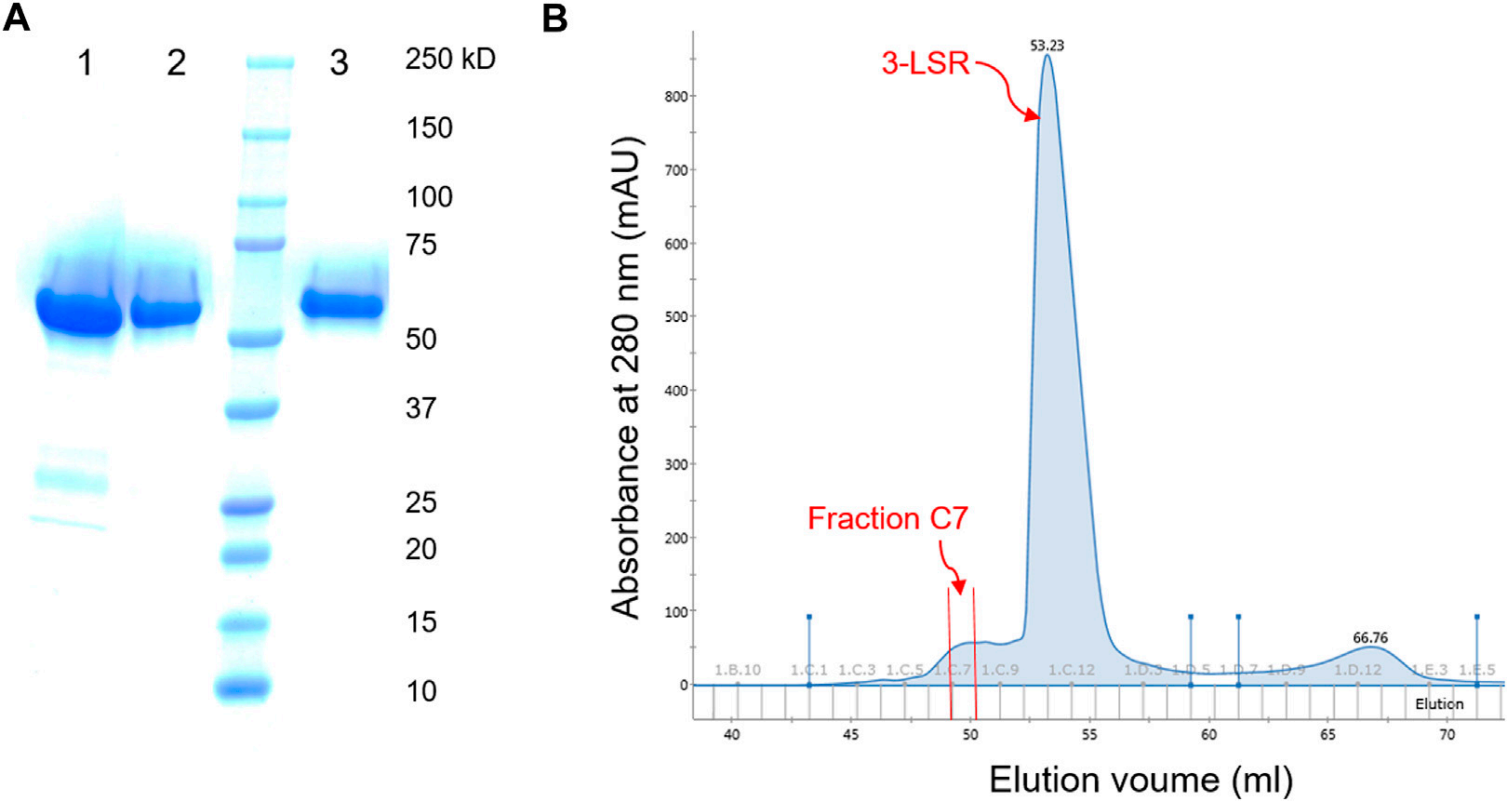
**(A)** SDS-PAGE of the 3-LSR after purification by Histrap (lane 1), anion-exchange (lane 2) and size exclusion (lane 3) chromatography. **(B)** Chromatogram of anion exchange purification of 3-LSR; the fraction with Flavin reductase activity (Fraction C7) eluted before elution of the 3-LSR protein (the main peak).

In this study, the RebH variant 3-LSR enzyme was recombinantly produced in *E. coli* with a yield of 15 mg protein per liter culture. To our surprise, we discovered that the partially purified enzyme (purified by HisTrap affinity chromatography) was able to catalyze bromination of 5-nitroindole (**1a**) without addition of any external Fre (Table 1 entry 1) forming the monobrominated product **2a** in high HPLC yields and selectivity. Remarkably, no multibrominated products were formed as observed by LC-MS analysis. To further investigate, the enzyme was purified to homogeneity with a final yield of 8.4 mg pure enzyme per liter culture using anion exchange and size exclusion chromatography (Figure 2A), and the purified enzyme was found to be almost inactive if no additional Fre was added (Table 1 entry 2). However, the activity of the purified enzyme was restored by addition of the flavin reductase enzyme RebF. The activity of the partially purified enzyme without external Fre was comparable to that of the purified enzyme supplemented with 5 µM RebF (Table 1 entry 3). To detect the anticipated co-purified Fre in the partially purified enzyme, small peaks from ion exchange and size exclusion fractions were tested for the reductase activity by using them as Fre source in the purified 3-LSR catalyzed halogenation. The purified enzyme was able to catalyze the halogenation reaction when supplemented with the fraction C7 from the ion exchange chromatography (Table 1 entry 4, Figure 2B). There was a small shift in retention time of the monobrominated product **2a** in HPLC for the reactions demonstrated in Table 1 as the samples were analyzed on different days thereby causing small variations in operational conditions e.g. pressure, flow, temperature and solvent ratios. To clarify further, the individual product peaks were confirmed by MS analysis. As illustrated in Supplementary Figure S9, the MS (ESI-) of the peak corresponding to **2a** in all the entries in Table 1 showed m/z of 239 and 241 with a typical isotope pattern of monobrominated compounds. Another set of MS peaks at m/z 353 and 355 was also observed which corresponds to TFA salt of **2a** as confirmed by HRMS (Supplementary Figure S26). No multibrominated products were detected by MS extraction of the corresponding masses.

**TABLE 1:** Bromination of 5-nitroindole with RebH 3-LSR.

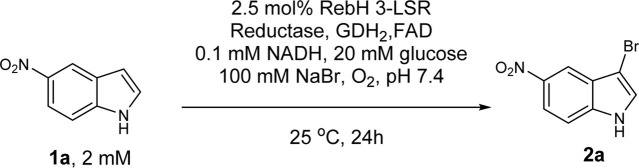				
**Entry**	**Halogenase used**	**Reductase added**	**HPLC yield (%)** ^[a]^	**Rentention time (min)** ^[b]^ **of 2a**
1	Partially purified 3-LSR^[c]^	None	97.7 ± 2.5	4.640
2	Purified 3-LSR^[d]^	None	6.3 ± 4.5	4.653
3	Purified 3-LSR^[d]^	5 µM RebF	95.6 ± 1.2	4.572
4	Purified 3-LSR^[d]^	50 µL fraction C7	96.5 ± 3.5	4.654
5	Purified 3-LSR^[d]^	5 µM purified AhpF	96.3 ± 1.5	4.571

[a] calculated from the HPLC peak area% of starting material and product; [b] A shift (up to ±0.08 min) in the retention time of **2a** in HPLC was observed for samples run on different days due to small variations in operational conditions-The product peaks for entries 1-5 were further confirmed by MS, analysis and illustrated in Supplementary Figure S9) [c] Purified by HisTrap affinity chromatography only; [d] Purified by HisTrap, ion-exchange and size exclusion chromatography.

Remarkably, mass-spectrometry analysis of fraction C7 identified an *E. coli* endogenous flavin reductase ‘alkyl hydroperoxide reductase F’ (AhpF) as the potential enzyme for FADH_2_ regeneration in the 3-LSR catalyzed halogenation reactions (Supplementary Figure S1). Proteomics analysis of the C7 fraction revealed 4 unique peptides covering 12.88% of the total protein sequences for AhpF (Table 2). AhpF is one of the ten most abundant proteins in *E. coli*, and is also present in other microorganisms (Link et al., 1997; Wood et al., 2001; Jiang and Bommarius, 2004; Dip et al., 2014). This homo-dimeric protein was also reported to be co-purified with other recombinant *E. coli* proteins (Haley, 2013; Kamariah et al., 2015; Klein et al., 2020). Its crystal structure consists of an FAD-binding domain and an NADH binding domain, and this protein is also known to have NADH-dependent FAD reductase activity (Dip et al., 2014; Kamariah et al., 2015). However, isolated AhpF was never used as Fre in FDH catalyzed reactions although it was reported to reduce free FAD to FADH_2_ *in vitro* (Jiang and Bommarius, 2004; Dip et al., 2014). We recombinantly expressed and purified the *E. coli* AhpF enzyme (Supplementary Figure S42) with a yield of 12 mg pure enzyme per liter culture. Activity of the purified 3-LSR enzyme in presence of 5 µM recombinant AhpF was comparable to that of the purified 3-LSR supplemented with 5 µM RebF (Table 1 entry 5), which suggests co-purified *E. coli* AhpF was acting as Fre in the partially purified enzyme preparations.

**TABLE 2:** Identification of AhpF protein from proteomics analysis of C7 fraction from anion exchange purification of 3-LSR. The sequence highlighted in yellow shows the coverage of the total protein sequences for AhpF. PSM represents the total peptide to spectrum match.

Total peptide	4	
Unique peptide	4	
Coverage (%)	12.88	
**Annotated peptide sequence**	**Sequence positions**	**PSM**
K.HTAIDGGTFQNEIIDR.N	150–165	1
K.VHVDEYDVDVIDSQSASK.L	274–291	2
K.LIPAAVEGGLHQIETASGAVLK.A	292–313	11
R.NMNVPGEDQYR.T	327–337	59
**>AhpF** LDTNMKTQLKAYLEKLTKPVELIATLDDSAKSAEIKELLAEIAELSDKVTFKEDNSLPVRKPSFLITNPGSNQGPRFAGSPLGHEFTSLVLALLWTGGHPSKEAQSLLEQIRHIDGDFEFETYYSLSCHNCPDVVQALNLMSVLNPRIK HTAIDGGTFQNEITDR NVMGVPAVFVNGKEFGQGRMTLTEIVAKIDTGAEKRAAEELNKRDAYDVLIVGSGPAGAAAAIYSARKGIRTGLMGERFGGQILDTVDIENYISVPKTEGQKLAGALK VHVDEYDVDVIDSQSASKLIPAAVEGGLHQIETASGAVLK ARSIIVATGAKWR NMNVPGEDQYR TKGVTYCPHCDGPLFKGKRVAVIGGGNSGVEAAIDLAGIVEHVTLLEFAPEMKADQVLQDKLRSLKNVDIILNAQTTEVKGDGSKVVGLEYRDRVSGDIHNIELAGIFVQIGLLPNTNWLEGAVERNRMGEIIIDAKCETNVKGVFAAGDCTTVPYKQIIIATGEGAKASLSAFDYLIRTKTA		

**FIGURE 3:**
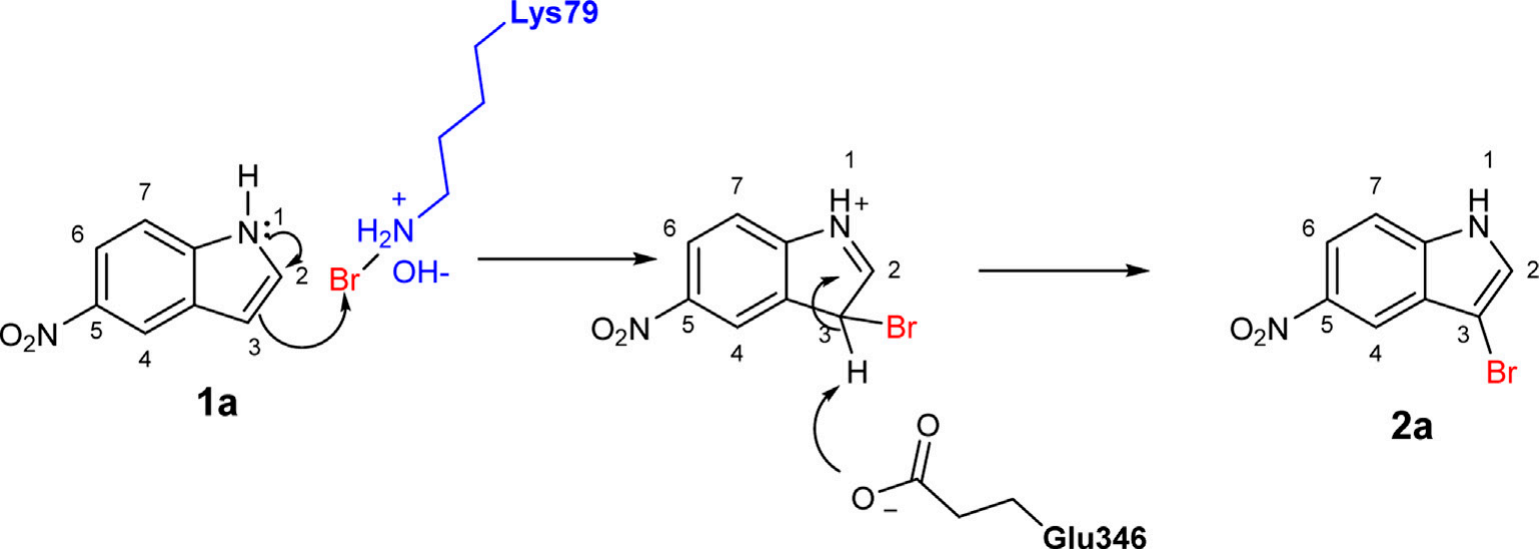
Proposed mechanism of 3-LSR catalyzed indole halogenation

To explore the scope of enzymatic bromination, the partially purified 3-LSR was used to brominate a panel of substrates (**1a**-**1o**) in a preparative scale as shown in Table 3. For indole halogenation, substituents at different positions on the benzenoid ring were well-tolerated by 3-LSR as monobrominated indoles were obtained in all cases as determined by LC-MS analysis. NMR analysis confirmed the formation of 3-bromoindoles (Supplementary Figure S2) (Sana et al., 2021). In analogy to the catalytic mechanism of tryptophan halogenases, the mechanism here is proposed to involve a Lys79-bromoamine intermediate active species which carries out electrophilic aromatic substitution at the most electron rich C3-position of the indole ring followed by deprotonation of the Wheland intermediate assisted by glutamate 346 oxygen to form 3-bromoindoles (Figure 3) (Fraley et al., 2017; Karabencheva-Christova et al., 2017; Schnepel and Sewald, 2017; Ismail et al., 2019). Electron rich indoles, such as 5-methoxyindole (**1e)** and 7-methylindole (**1k**) were brominated with good to high yields. Interestingly, indole derivatives containing electron withdrawing substituents such as 5-nitroindole (**1a**), methyl indole-5-carboxylate (**1d**) and halogen substituted indoles (**1f-1h**, **1j** and **1m**) were also effective substrates showing moderate to high yields of the corresponding monobrominated products. Carboxylic functionalities were well tolerated as shown by the examples **1c, 1d** and **1i**. Azaindoles, which serve as isosteres of indoles in medicinal chemistry, were brominated efficiently despite the presence of a π-electron deficient pyridine ring (**1l-n**). This indicates that the propensity of bromination is not simply determined by substitution kinetics but there are other factors at play, for example, interactions between indole and the active site of halogenase. NMR analysis identified C3 position as the halogenation site of the azaindole compounds. Chlorination of **1a** was also achieved in a high yield when NaBr was replaced by NaCl (entry 16). Remarkably, the anthranilamide derivative **1o** was efficiently brominated by 3-LSR to give 2-amino-5-bromobenzamide (**2o**) in high selectivity. It is noteworthy that di- or tri-halogenated products were not observed in any of these cases as confirmed by LC-MS analysis.

**TABLE 3:** Halogenation of indoles and other substrates by partially purified 3-LSR.

Entry	Substrates	Products (HPLC yield, %)^[a]^
1	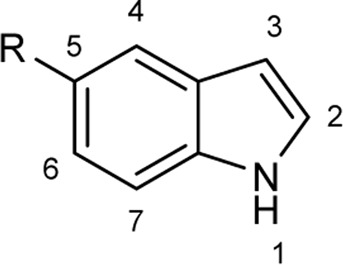 R = NO_2_, **1a**	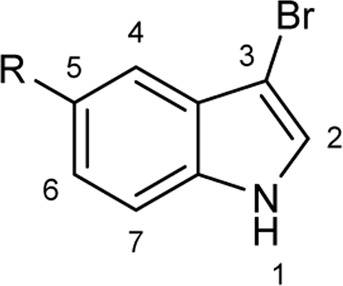 <**2a**, >99
2	R = CN, **1b**	**2b**, 55
3	R = CO_2_H, **1c**	**2c**, 33
4	R = CO_2_Me, **1d**	**2d**, 86
5	R = OMe, **1e**	**2e**, 40
6	R = Br, **1f**	2f*, 48
7	R = Cl, **1g**	2g*, 38
8	R = F, **1h**	2h*, 30
9	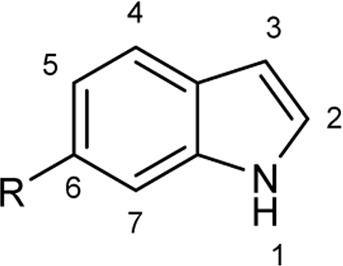 R = CO_2_H, **1i**	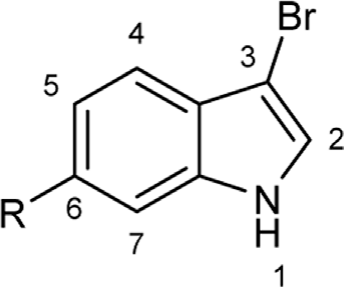 **2i**, 49
10	R = F, **1j**	2j*, 83
11	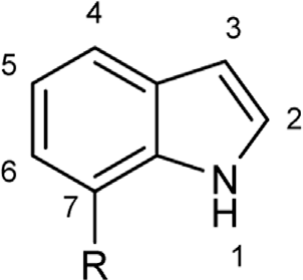 R = Me, **1k**	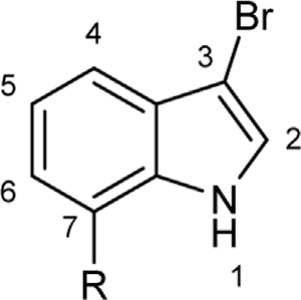 **2k**, 82
12	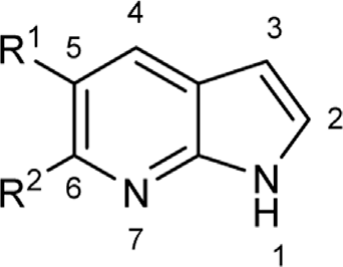 R^1^ = R^2^ = H, **1l**	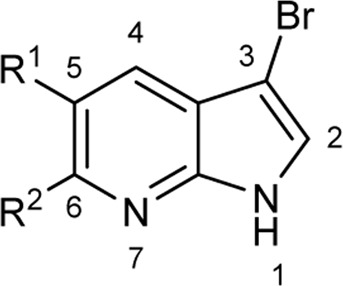 **2l**, 98
13	R^1^ = Br, R^2^ = H, **1m**	2m*, 14
14	R^1^ = H, R^2^ = OMe, **1n**	2n*, 98
15	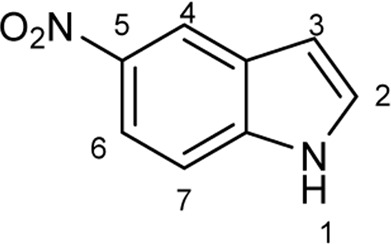 **1a**	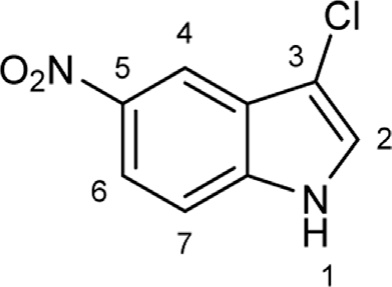 **2a'**, 99
16	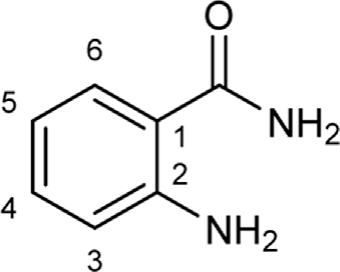 **1o**	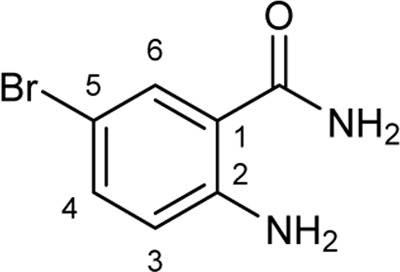 **2o**, >99

[a] calculated from the HPLC peak area% of starting material and product. Only mono halogenated products were observed. * These products were not isolated.

In conclusion, we have established a panel of indole, azaindole and anthranilamide compounds that are substrates for the thermostable RebH variant 3-LSR. Indoles and azaindoles without any substituents at C2/C3 positions were brominated at the most electrophilic site to give 3-bromoindole derivatives. Interestingly, high conversion levels were achieved using partially purified 3-LSR without addition of any external flavin reductase enzyme. Further investigation confirmed that AhpF, an endogenous *E. coli* flavin reductase enzyme co-purified with the 3-LSR, and catalyzed the NADH-dependent FAD regeneration for constant supply of the co-factor FADH_2_ essential for FDH catalyzed halogenation reactions. The co-purification of AhpF with the halogenase enzyme 3-LSR simplifies the overall catalytic process eliminating the need for additional production and purification of Fre enzyme.

## Data Availability

The mass spectrometry data in this study are deposited in the MassIVE repository, accession number MSV000090619.
