# Supplementary material for: Indole and azaindole halogenation catalyzed by the RebH enzyme variant 3-LSR utilizing co-purified E. coli reductase
Source: Front Bioeng Biotechnol. 2022 Dec 1;10:1032707. doi: 10.3389/fbioe.2022.1032707 (PMC9801302; doi:10.3389/fbioe.2022.1032707)
Supplement: Supplementary file 1 [file DataSheet1.pdf]

Supplementary Material

Indole and Azaindole Halogenation Catalyzed by the RebH Enzyme Variant 3-LSR Utilizing Co-Purified *E. coli* Reductase

Eunice Hui Yen Li[1†], Barindra Sana[2†], Timothy Ho[1], Ding Ke[2], Farid J Ghadessy[2*], Hung A Duong[1*], Jayasree Seayad[1*]

[1]Institute of Sustainability for Chemicals, Energy and Environment, A*STAR, 8 Biomedical Grove, Neuros, #07-01, Singapore 138665

[2]Disease Intervention Technology Laboratory, Institute of Molecular and Cellular Biology, A*STAR, 8A Biomedical Grove, #06-04/05 Neuros/Immunos, Singapore 138648

†These authors contributed equally to this work and share first authorship

*** Correspondence:**
Farid J Ghadessy
fghadessy@imcb.a-star.edu.sg
Duong Anh Hung
duonganhhung@hotmail.com
Jayasree Seayad
jayasree_seayad@isce2.a-star.edu.sg

1 Supplementary Data

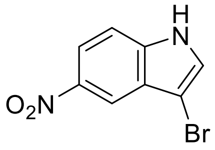

3-bromo-5-nitro-1H-indole (2a)[1]

Isolated yield: 0.4 mg (26%). ^{1}H NMR (CD_3OD, 400 MHz) δ (ppm) 8.43 (d, *J* = 2.2 Hz, 1H), 8.11 (dd, *J* = 9.0, 2.3 Hz, 1H), 7.59 – 7.51 (m, 2H). ^{13}C NMR (CD_3OD, 101 MHz) δ (ppm) 143.39, 140.21, 129.19, 127.58, 118.74, 116.54, 113.33, 93.06. HRMS (ESI): m/z $[M-H]^-$ calcd for $C_8H_5{}^{79}BrN_2O_2$ 238.9461; found 238.9451. HRMS (ESI): m/z $[M+CF_3COO]^-$ calcd for $C_{10}H_5{}^{79}BrF_3N_2O_4$ 352.9385; found 352.9379.

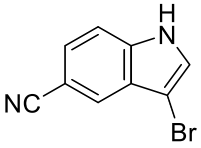

3-bromo-1H-indole-5-carbonitrile (2b)[2]

Isolated yield: 0.5 mg (32%). ^{1}H NMR (CD_3OD, 400 MHz) δ (ppm) 7.87 (dd, *J* = 1.6, 0.7 Hz, 1H), 7.57 – 7.44 (m, 3H). ^{13}C NMR (CD_3OD, 101 MHz) δ (ppm) 139.0, 128.2, 128.1, 126.1, 125.2, 121.2, 114.2, 104.0, 91.6. HRMS (ESI): m/z $[M-H]^-$ calcd for $C_9H_5{}^{79}BrN_2$: 218.9563; found 218.9555.

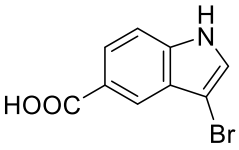

3-bromo-1H-indole-5-carboxylic acid (2c)[2]

Isolated yield: 0.1 mg (9%). ^{1}H NMR (CD_3OD, 400 MHz) δ (ppm) 8.22 (d, *J* = 1.6 Hz, 1H), 7.87 (dd, *J* = 8.6, 1.6 Hz, 1H), 7.44 – 7.37 (m, 2H). HRMS (ESI): m/z $[M-H]^-$ calcd for $C_9H_6{}^{79}BrNO_2$ 237.9509; found 237.9499.

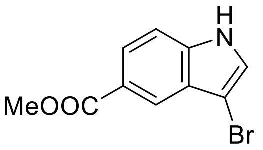

Methyl 3-bromo-1H-indole-5-carboxylate (2d)[3]

Isolated yield: 0.9 mg (65%). ^{1}H NMR (CD_3OD, 400 MHz) δ (ppm) 8.21 (s, 1H), 7.86 (d, *J* = 8.7 Hz, 1H), 7.48 – 7.40 (m, 2H), 3.93 (s, 3H). HRMS (ESI): m/z $[M-H]^-$ calcd for $C_{10}H_8{}^{79}BrNO_2$ $[M-H]^-$ 251.9665; found 251.9655.

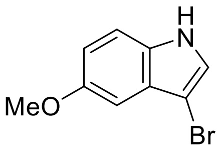

3-bromo-5-methoxy-1H-indole (2e)[4]

Isolated yield: 1.0 mg (65%). ^{1}H NMR (CD_3OD, 400 MHz) δ (ppm) 7.29 – 7.21 (m, 2H), 6.90 (d, *J* = 2.5 Hz, 1H), 6.82 (dd, *J* = 8.8, 2.4 Hz, 1H), 3.83 (s, 3H). HRMS (ESI): m/z $[M-H]^-$ calcd for $C_9H_8{}^{79}BrNO$ 223.9716; found 223.9713.

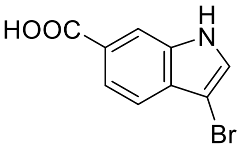

3-bromo-1H-indole-6-carboxylic acid (2i)

Isolated yield: 1.0 mg (67%). ^{1}H NMR (CD_3OD, 400 MHz) δ (ppm) 8.15 – 8.12 (m, 1H), 7.80 (dd, *J* = 8.4, 1.4 Hz, 1H), 7.53 – 7.47 (m, 2H). ^{13}C NMR (CD_3OD, 101 MHz) δ (ppm) 171.09, 136.45, 131.36, 128.80, 126.01, 122.09, 119.02, 115.39, 91.12. HRMS (ESI): m/z $[M-H]^-$ calcd for $C_9H_6{}^{79}BrNO_2$ 237.9509; found 237.9499.

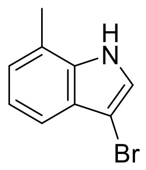

3-bromo-7-methyl-1H-indole (2k)[5]

Isolated yield: 0.4 mg (24%). ^{1}H NMR (CD_3OD, 400 MHz) δ (ppm) 7.48 – 7.44 (m, 1H), 7.14 (d, *J* = 1.6 Hz, 1H), 6.92 – 6.83 (m, 2H), 2.44 (d, *J* = 0.9 Hz, 3H). MS (EI): m/z [M] calcd. for $C_9H_8{}^{79}BrN$ 209; found 209.

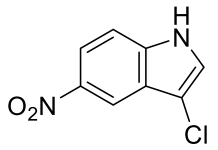

3-chloro-5-nitro-1H-indole (2a')[6]

Isolated yield: 0.8 mg (52%). ^{1}H NMR (CD_3OD, 400 MHz) δ (ppm) 8.49 (dd, *J* = 2.3, 0.5 Hz, 1H), 8.11 (dd, *J* = 9.0, 2.3 Hz, 1H), 7.56 – 7.48 (m, 2H). HRMS (ESI): m/z $[M-H]^-$ calcd for $C_8H_5ClN_2O_2$ 194.9967; found 194.9957.

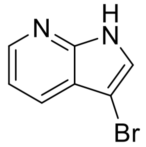

3-bromo-1H-pyrrolo[2,3-b]pyridine (2l)[2]

Isolated yield: 1.4 mg (84%). ^{1}H NMR (CD_3OD, 400 MHz) δ (ppm) 8.26 (dd, *J* = 4.8, 1.6 Hz, 1H), 7.92 (dd, *J* = 7.9, 1.5 Hz, 1H), 7.47 (s, 1H), 7.20 (dd, *J* = 7.9, 4.8 Hz, 1H). HRMS (ESI): m/z $[M+H]^+$ calcd for $C_7H_5{}^{79}BrN_2$ 196.9709; found 196.9706.

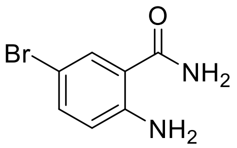

2-amino-5-bromobenzamide (2o)[7]

Isolated yield: 1.4 mg (90%). ^{1}H NMR (($CD_3)_2SO$, 400 MHz) δ (ppm) 7.83 (s, 1H), 7.69 (d, *J* = 2.4 Hz, 1H), 7.25 (dd, *J* = 8.8, 2.4 Hz, 1H), 7.16 (s, 1H), 6.70 (s, 2H), 6.65 (d, *J* = 8.8 Hz, 1H). ^{13}C NMR (($CD_3)_2SO$, 101 MHz) δ (ppm) 170.0, 149.4, 134.4, 130.8, 118.5, 115.2, 104.8. HRMS (ESI): m/z $[M+H]^+$ calcd for $C_7H_7{}^{79}BrN_2O$ 214.9815; found 214.9809.

2 Supplementary Figures

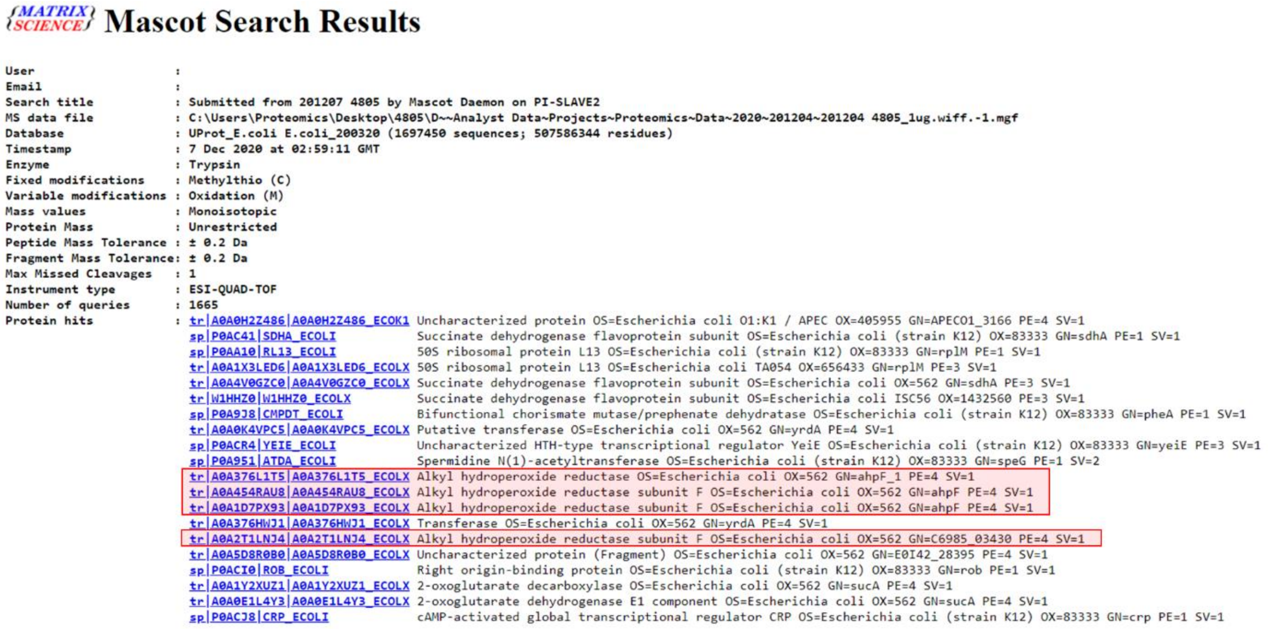

Mascot Search Results

```
User                    :
Email                   :
Search title            : Submitted from 201207 4805 by Mascot Daemon on PI-SLAVE2
MS data file            : C:\Users\Proteomics\Desktop\4805\D~~Analyst Data~Projects~Proteomics~Data~2020~201204~201204 4805_1ug.wiff.-1.mgf
Database                : UProt_E.coli E.coli_200320 (1697450 sequences; 507586344 residues)
Timestamp               : 7 Dec 2020 at 02:59:11 GMT
Enzyme                  : Trypsin
Fixed modifications     : Methylthio (C)
Variable modifications  : Oxidation (M)
Mass values             : Monoisotopic
Protein Mass            : Unrestricted
Peptide Mass Tolerance  : ± 0.2 Da
Fragment Mass Tolerance: ± 0.2 Da
Max Missed Cleavages    : 1
Instrument type         : ESI-QUAD-TOF
Number of queries       : 1665
Protein hits            : tr|A0A0H2Z486|A0A0H2Z486_ECOK1 Uncharacterized protein OS=Escherichia coli O1:K1 / APEC OX=405955 GN=APECO1_3166 PE=4 SV=1
                          sp|P0AC41|SDHA_ECOLI           Succinate dehydrogenase flavoprotein subunit OS=Escherichia coli (strain K12) OX=83333 GN=sdhA PE=1 SV=1
                          sp|P0AA10|RL13_ECOLI           50S ribosomal protein L13 OS=Escherichia coli (strain K12) OX=83333 GN=rplM PE=1 SV=1
                          tr|A0A1X3LED6|A0A1X3LED6_ECOLX 50S ribosomal protein L13 OS=Escherichia coli TA054 OX=656433 GN=rplM PE=3 SV=1
                          tr|A0A4V0GZC0|A0A4V0GZC0_ECOLX Succinate dehydrogenase flavoprotein subunit OS=Escherichia coli OX=562 GN=sdhA PE=3 SV=1
                          tr|W1HHZ0|W1HHZ0_ECOLX         Succinate dehydrogenase flavoprotein subunit OS=Escherichia coli ISC56 OX=1432560 PE=3 SV=1
                          sp|P0A9J8|CMPDT_ECOLI          Bifunctional chorismate mutase/prephenate dehydratase OS=Escherichia coli (strain K12) OX=83333 GN=pheA PE=1 SV=1
                          tr|A0A0K4VPC5|A0A0K4VPC5_ECOLX Putative transferase OS=Escherichia coli OX=562 GN=yrdA PE=4 SV=1
                          sp|P0ACR4|YEIE_ECOLI           Uncharacterized HTH-type transcriptional regulator YeiE OS=Escherichia coli (strain K12) OX=83333 GN=yeiE PE=3 SV=1
                          sp|P0A951|ATDA_ECOLI           Spermidine N(1)-acetyltransferase OS=Escherichia coli (strain K12) OX=83333 GN=speG PE=1 SV=2
                          tr|A0A376L1T5|A0A376L1T5_ECOLX Alkyl hydroperoxide reductase OS=Escherichia coli OX=562 GN=ahpF_1 PE=4 SV=1
                          tr|A0A454RAU8|A0A454RAU8_ECOLX Alkyl hydroperoxide reductase subunit F OS=Escherichia coli OX=562 GN=ahpF PE=4 SV=1
                          tr|A0A1D7PX93|A0A1D7PX93_ECOLX Alkyl hydroperoxide reductase subunit F OS=Escherichia coli OX=562 GN=ahpF PE=4 SV=1
                          tr|A0A376HWJ1|A0A376HWJ1_ECOLX Transferase OS=Escherichia coli OX=562 GN=yrdA PE=4 SV=1
                          tr|A0A2T1LNJ4|A0A2T1LNJ4_ECOLX Alkyl hydroperoxide reductase subunit F OS=Escherichia coli OX=562 GN=C6985_03430 PE=4 SV=1
                          tr|A0A5D8R0B0|A0A5D8R0B0_ECOLX Uncharacterized protein (Fragment) OS=Escherichia coli OX=562 GN=E0I42_28395 PE=4 SV=1
                          sp|P0ACI0|ROB_ECOLI            Right origin-binding protein OS=Escherichia coli (strain K12) OX=83333 GN=rob PE=1 SV=1
                          tr|A0A1Y2XUZ1|A0A1Y2XUZ1_ECOLX 2-oxoglutarate decarboxylase OS=Escherichia coli OX=562 GN=sucA PE=4 SV=1
                          tr|A0A0E1L4Y3|A0A0E1L4Y3_ECOLX 2-oxoglutarate dehydrogenase E1 component OS=Escherichia coli OX=562 GN=sucA PE=4 SV=1
                          sp|P0ACJ8|CRP_ECOLI            cAMP-activated global transcriptional regulator CRP OS=Escherichia coli (strain K12) OX=83333 GN=crp PE=1 SV=1
```

Supplementary Figure S1. Mascot search results of the mass spectrum for fraction C7

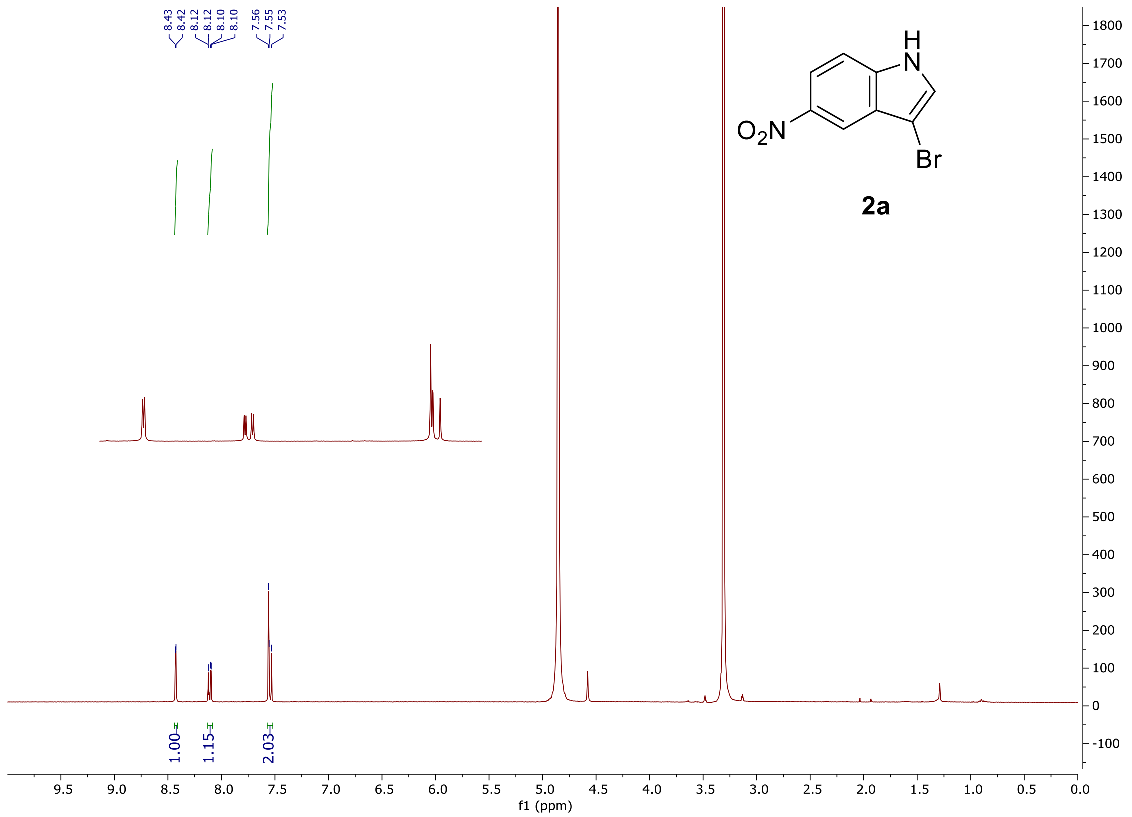

Supplementary Figure S2. ^{1}H Spectra of **2a**.

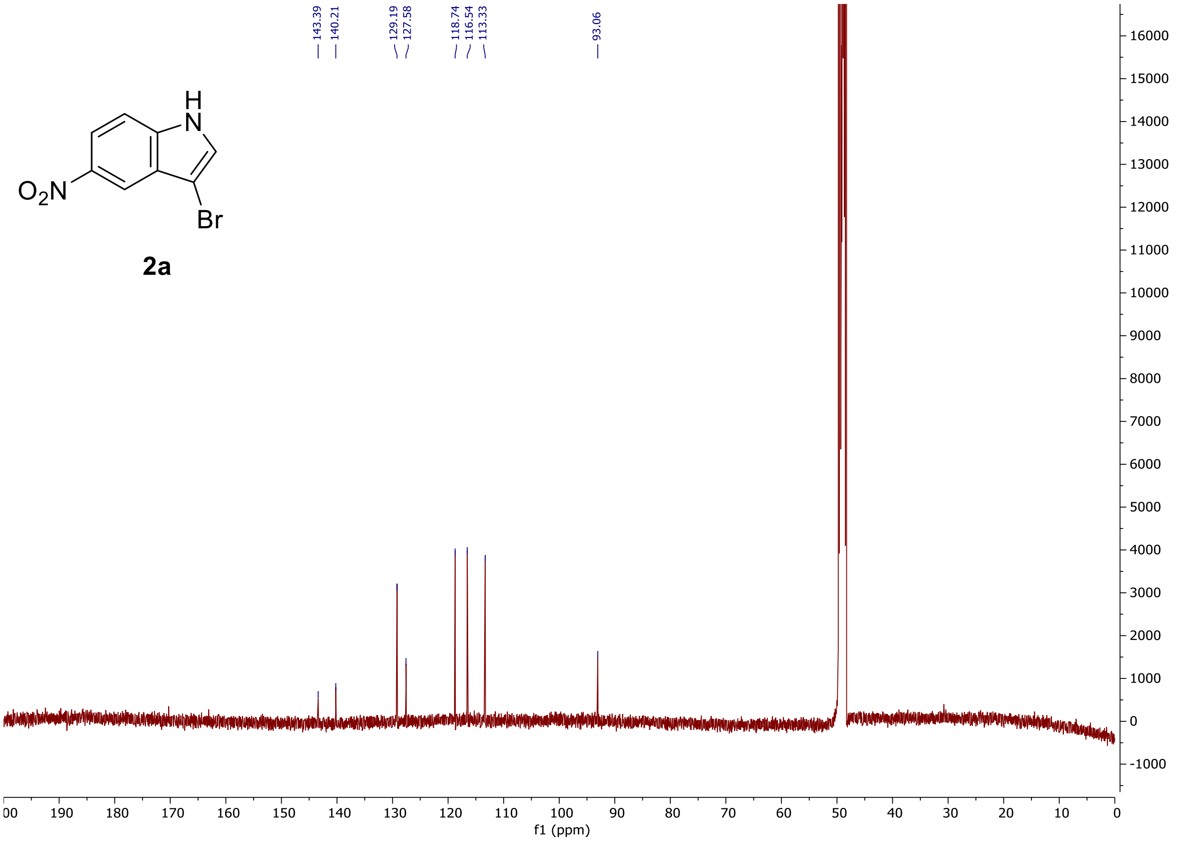

2a

Supplementary Figure S3. ^{13}C spectra of **2a**.

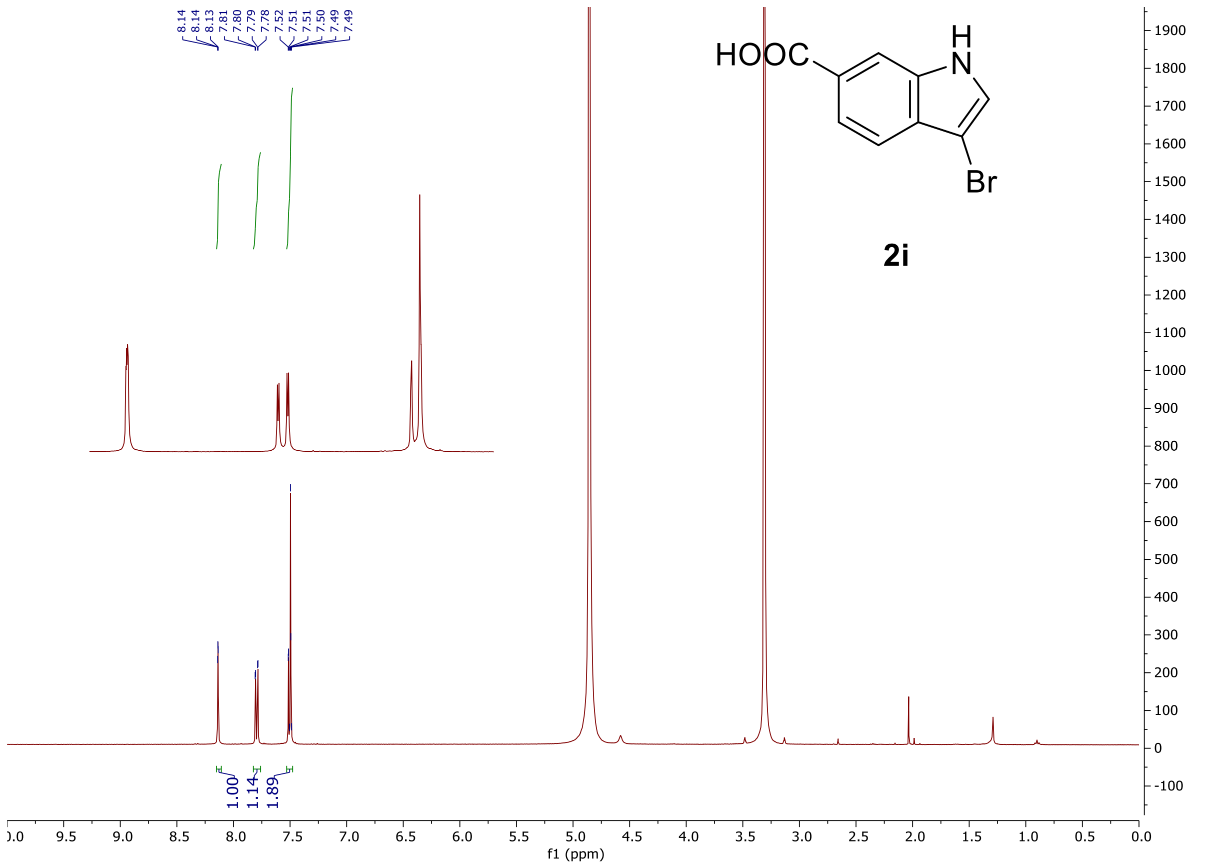

Supplementary Figure S4. ^{1}H Spectra of **2i**.

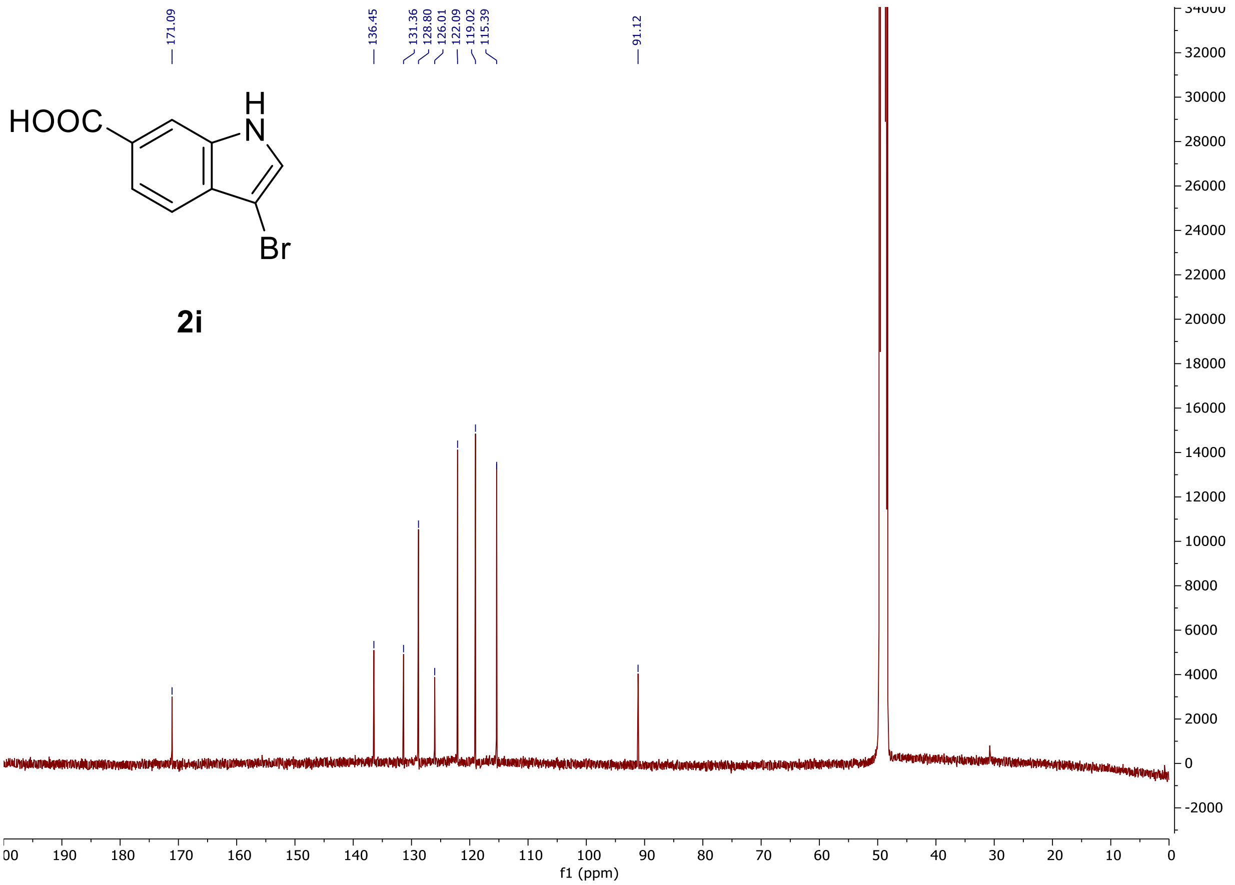

Supplementary Figure S5. ^{13}C Spectra of **2i**.

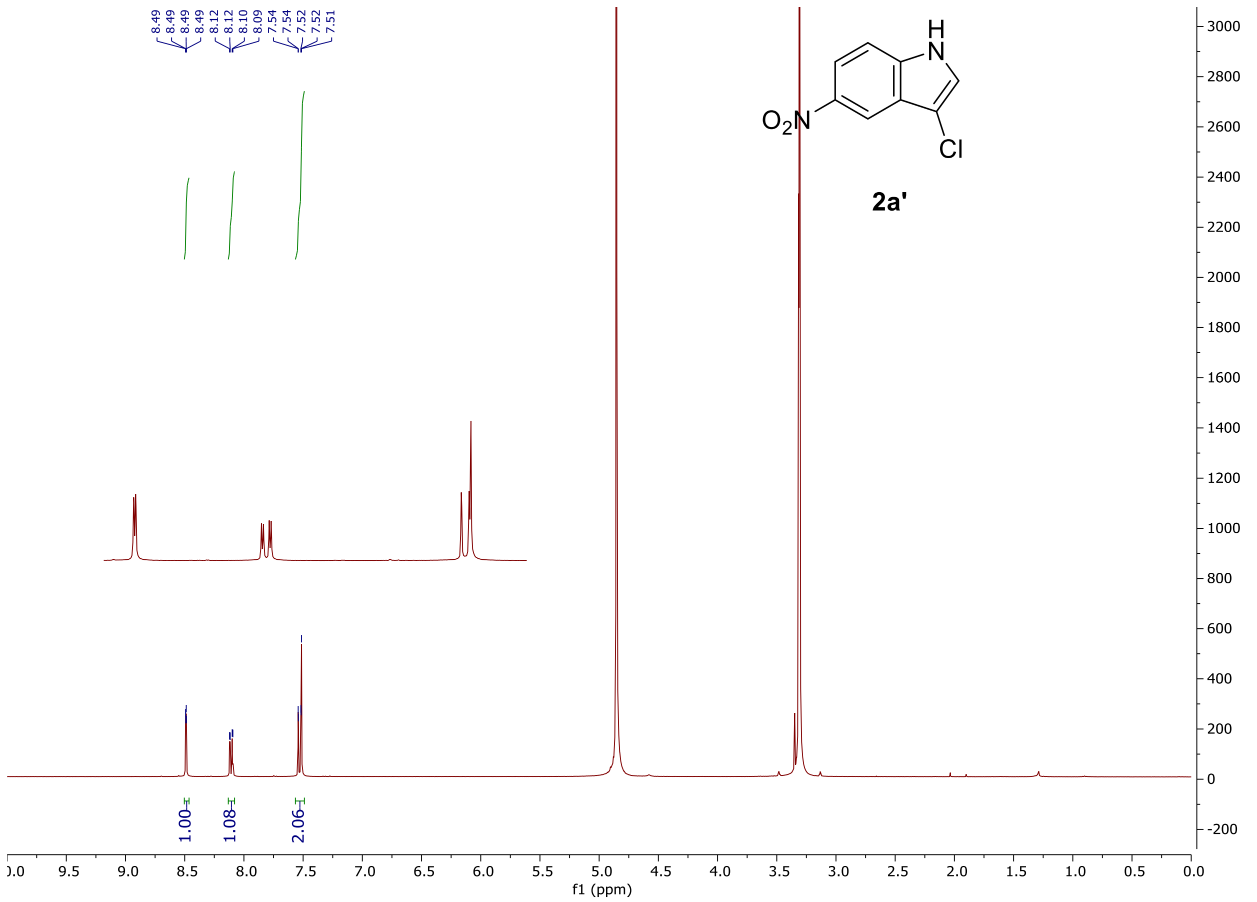

Supplementary Figure S6. ^{1}H Spectra of **2a’**.

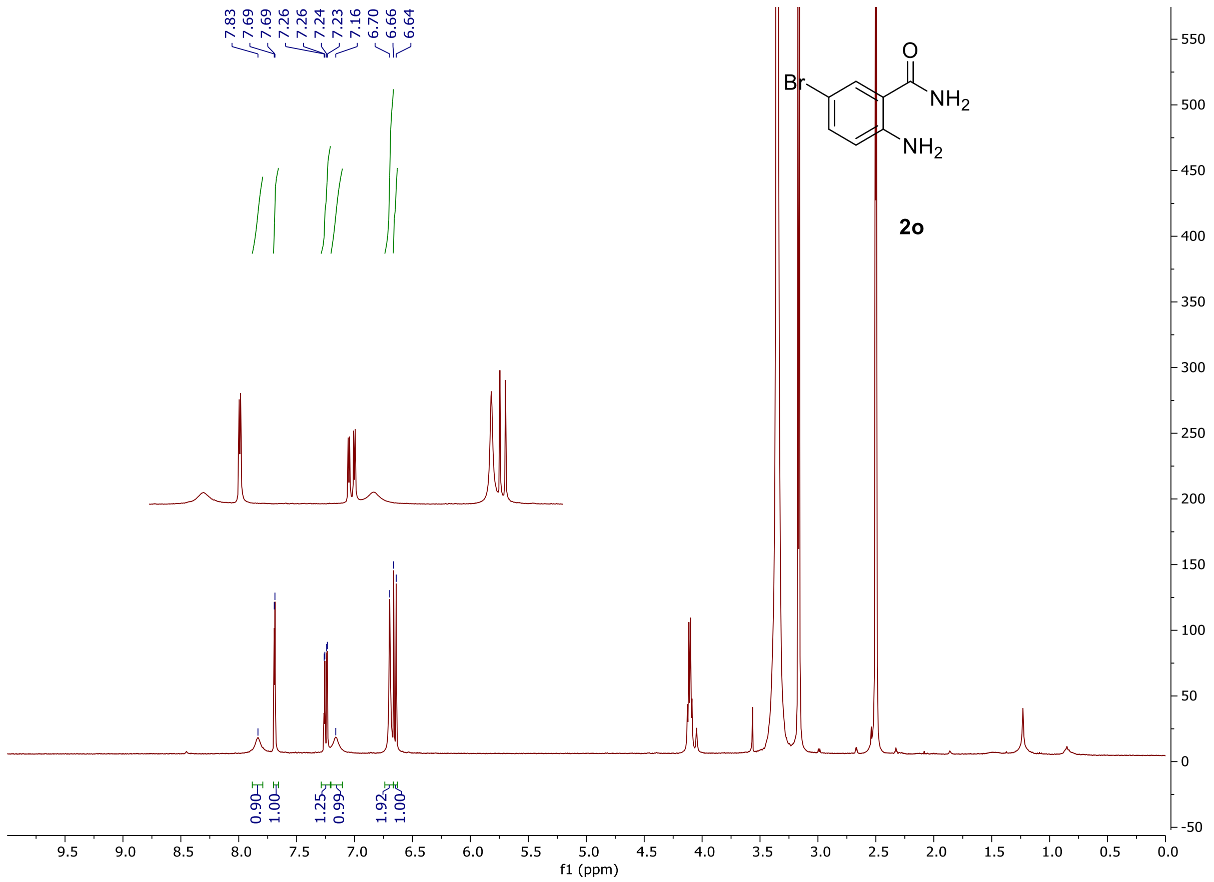

Supplementary Figure S7. [1]H Spectra of **2o.**

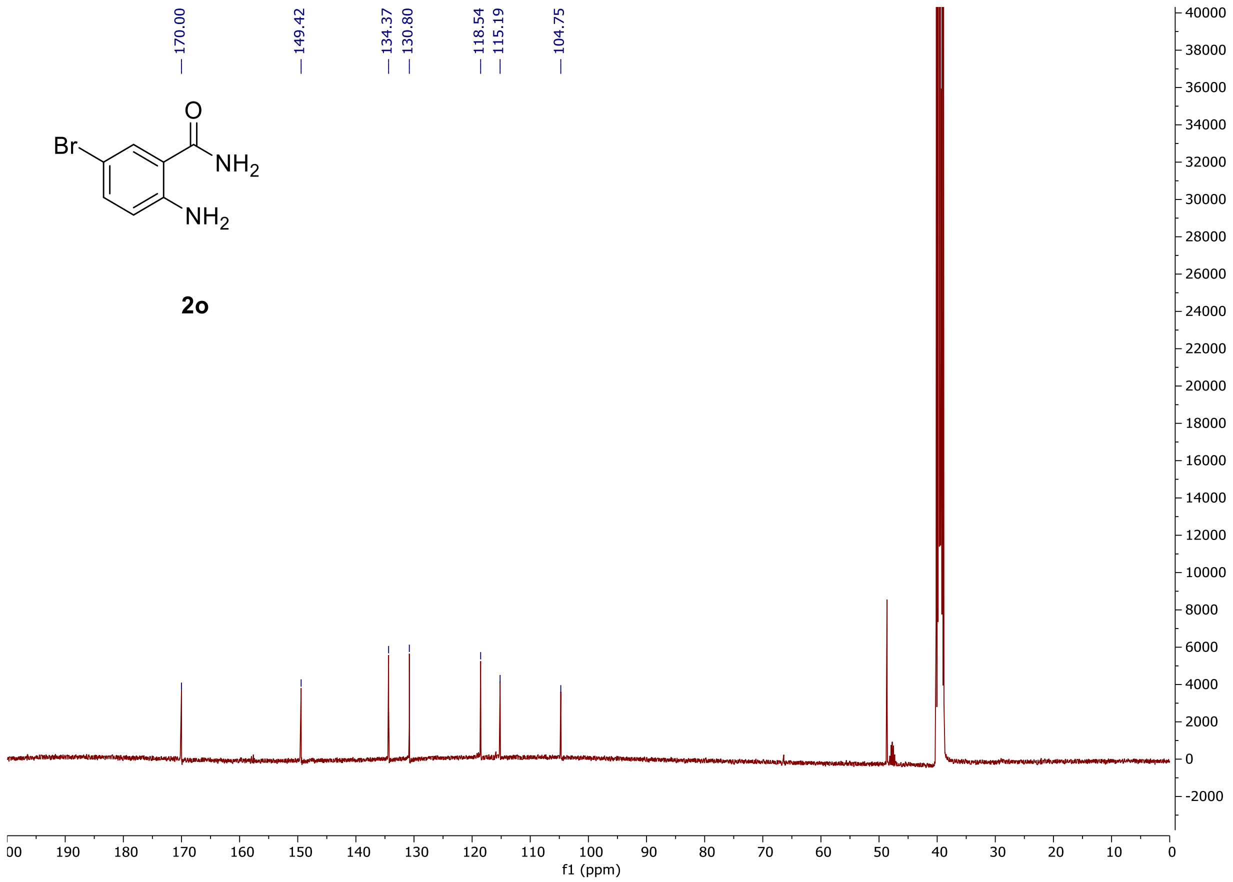

Supplementary Figure S8. ^{13}C Spectra of **2o**.

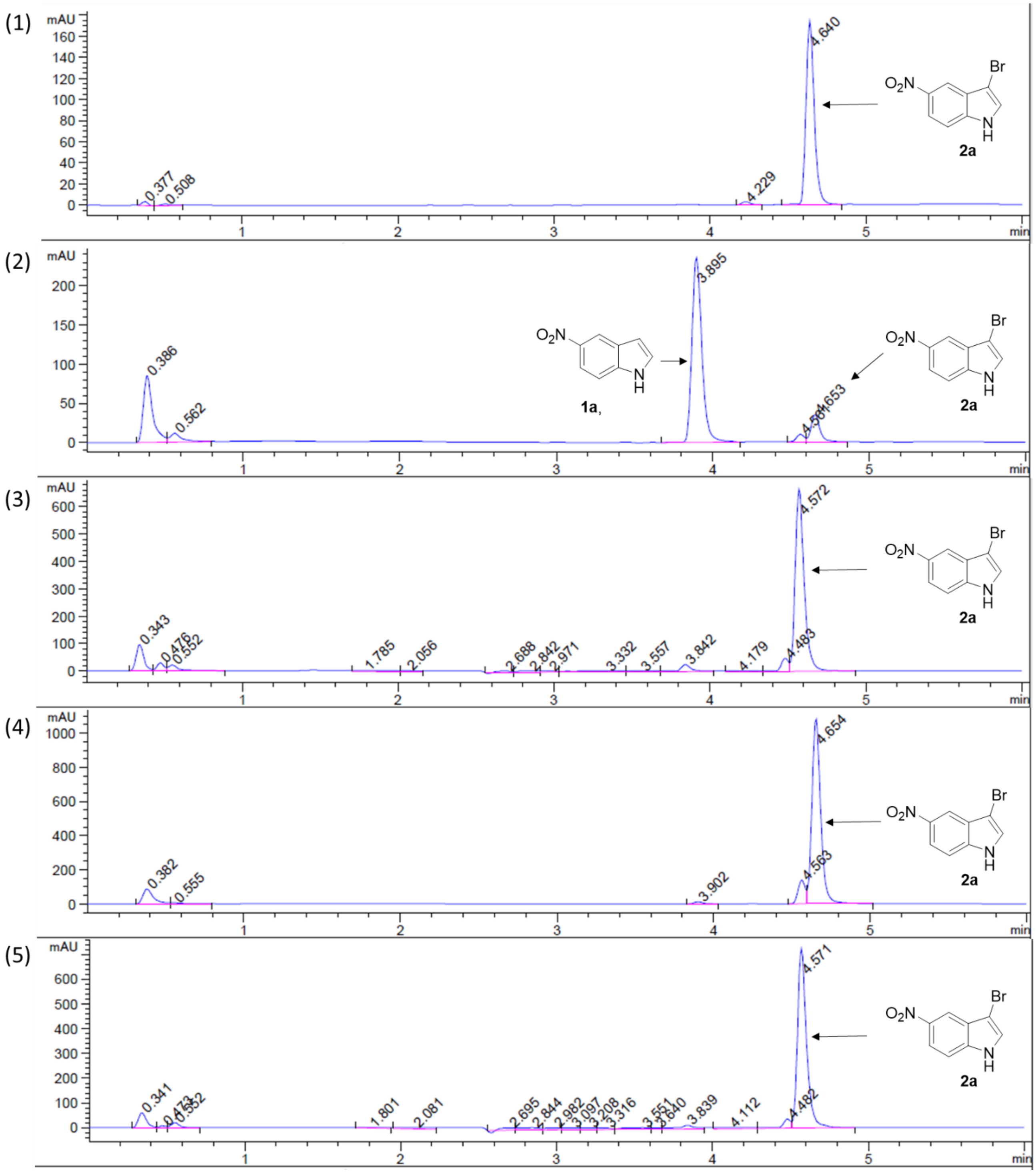
(1)
mAU
4.640
4.229
0.377
0.508
min
O_2N
Br
N
H
2a
(2)
mAU
3.895
0.386
0.562
4.653
1a,
2a
(3)
mAU
4.572
0.343
0.476
0.552
1.785
2.056
2.688
2.842
2.971
3.332
3.557
3.842
4.179
4.483
2a
(4)
mAU
4.654
4.563
3.902
0.382
0.555
2a
(5)
mAU
4.571
0.341
1.801
2.081
2.695
2.844
2.982
3.097
3.208
3.316
3.551
3.640
3.839
4.112
4.482
2a

Entry 1

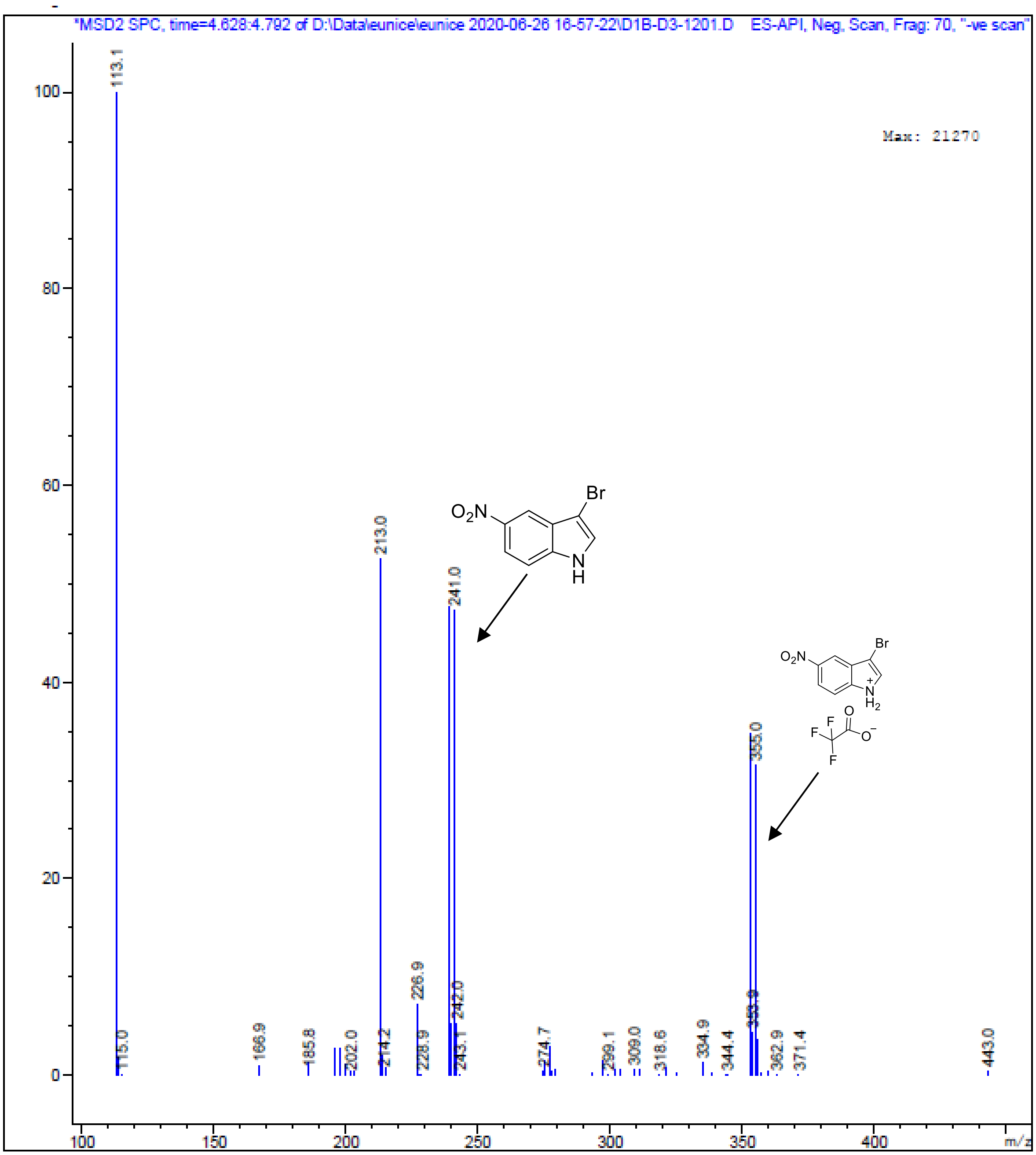
"MSD2 SPC, time=4.628:4.792 of D:\Data\eunice\eunice 2020-06-26 16-57-22\D1B-D3-1201.D ES-API, Neg, Scan, Frag: 70, "-ve scan"
Max: 21270
113.1
213.0
241.0
355.0
Br
O2N
N
H
F
O
m/z

Entry 2

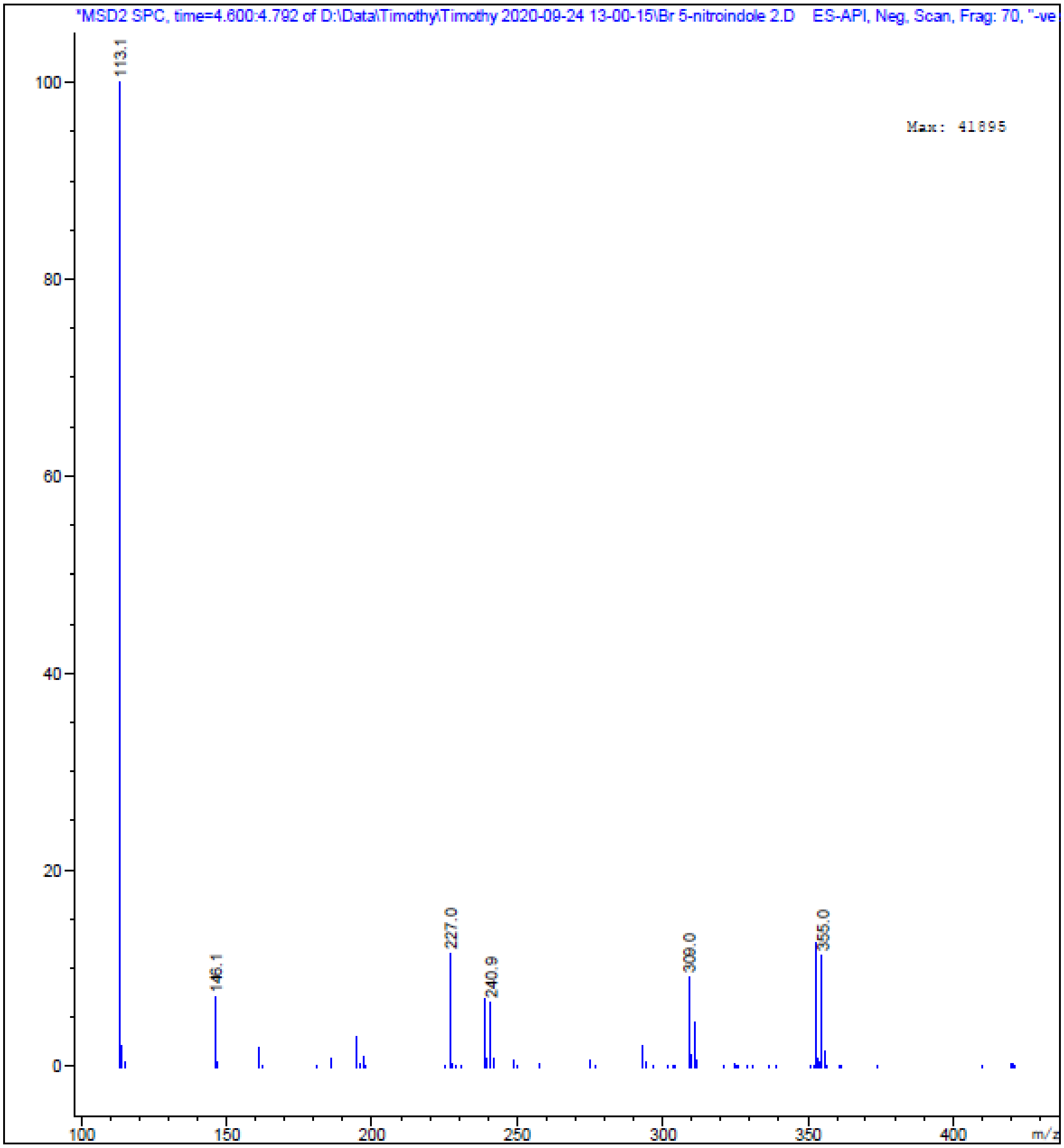

*MSD2 SPC, time=4.600:4.792 of D:\Data\Timothy\Timothy 2020-09-24 13-00-15\Br 5-nitroindole 2.D ES-API, Neg, Scan, Frag: 70, "-ve
Max: 41895
113.1
146.1
227.0
240.9
309.0
355.0
100
80
60
40
20
0
100
150
200
250
300
350
400
m/z

Entry 3

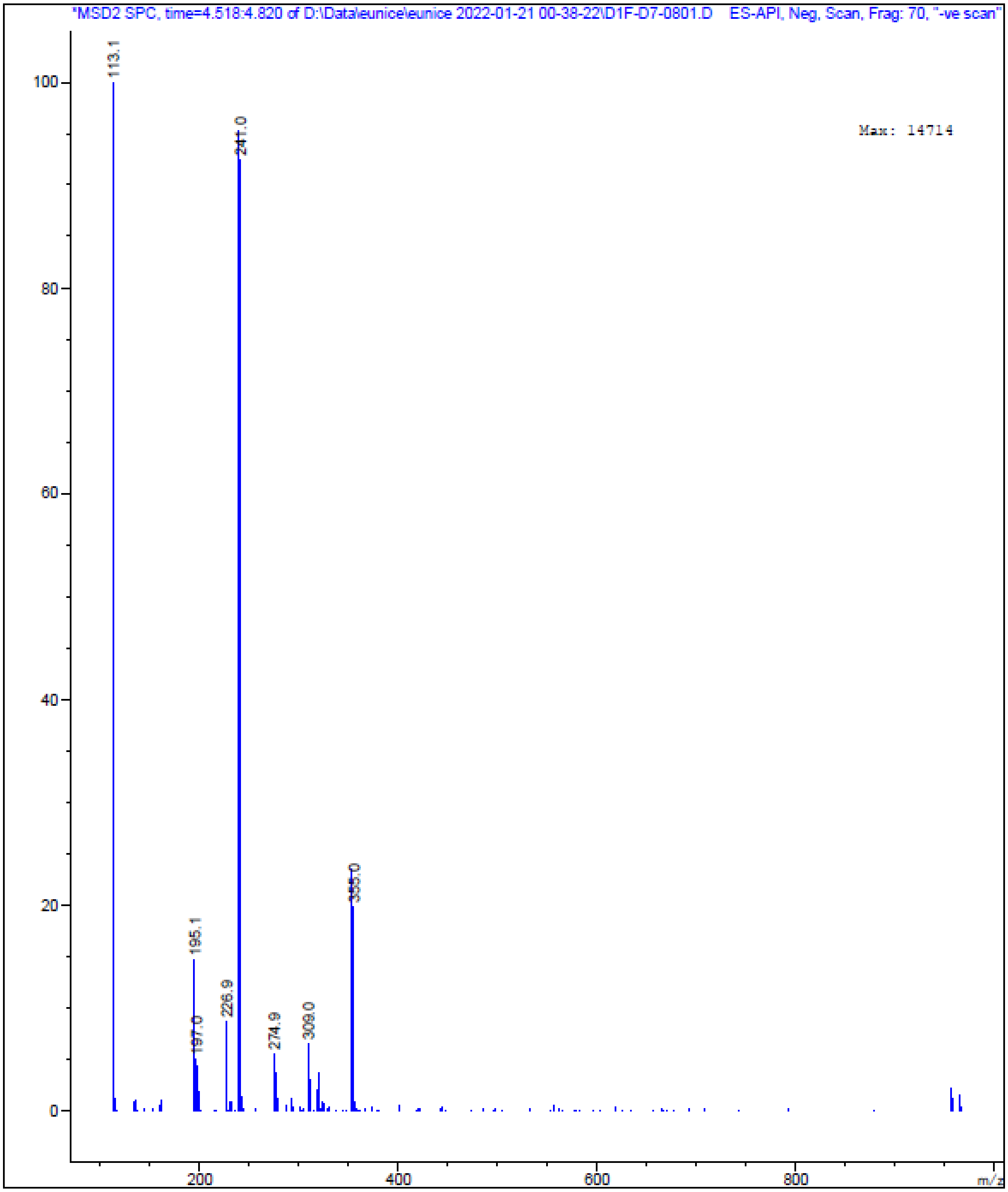
*MSD2 SPC, time=4.518:4.820 of D:\Data\eunice\eunice 2022-01-21 00-38-22\D1F-D7-0801.D ES-API, Neg, Scan, Frag: 70, "-ve scan"
Max: 14714
113.1
241.0
195.1
197.0
226.9
274.9
309.0
355.0
100
80
60
40
20
0
200
400
600
800
m/z

Entry 4

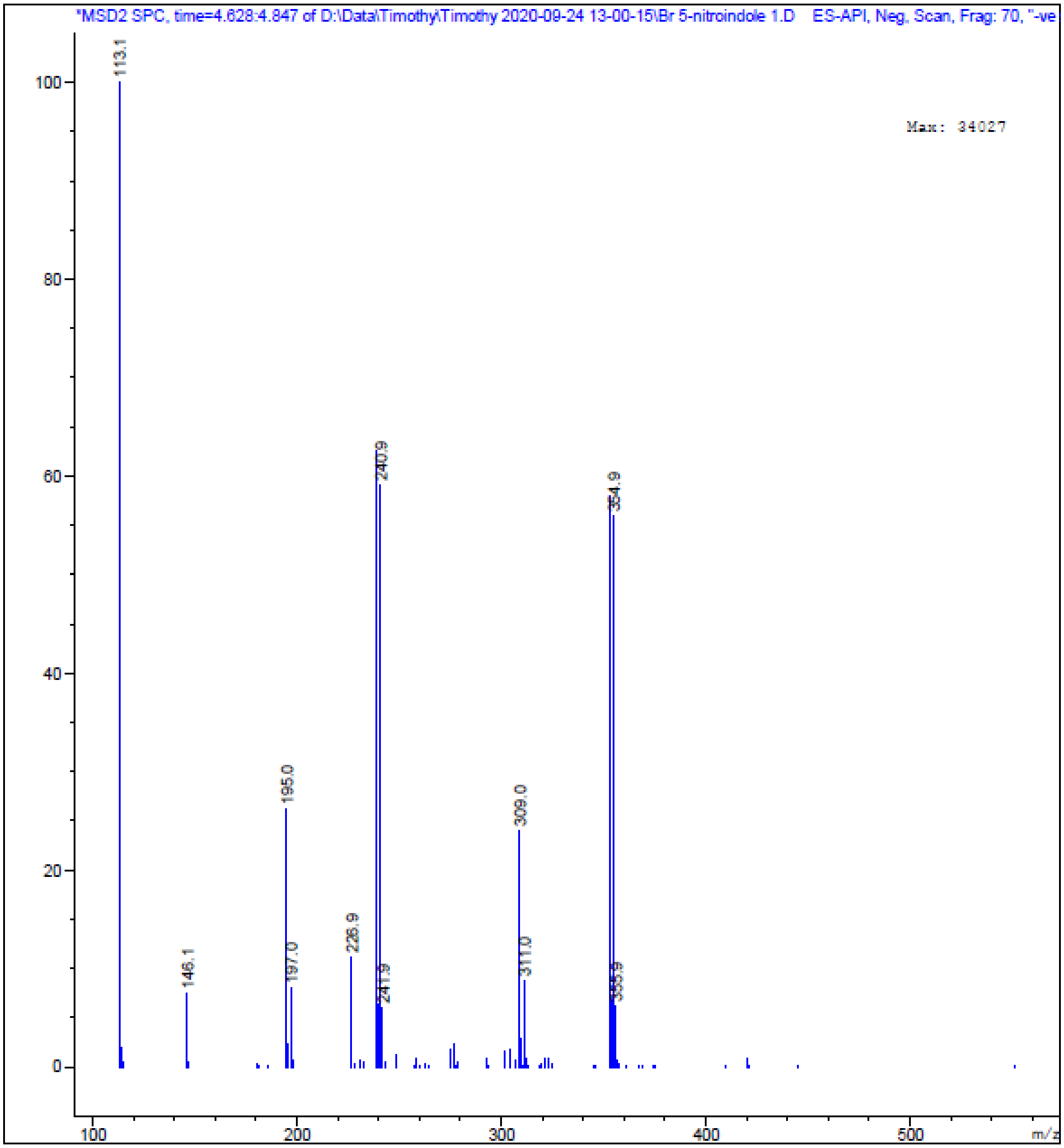
*MSD2 SPC, time=4.628:4.847 of D:\Data\Timothy\Timothy 2020-09-24 13-00-15\Br 5-nitroindole 1.D ES-API, Neg, Scan, Frag: 70, "-ve
Max: 34027
113.1
146.1
195.0
197.0
226.9
240.9
241.9
309.0
311.0
354.9
355.9
m/z

Entry 5

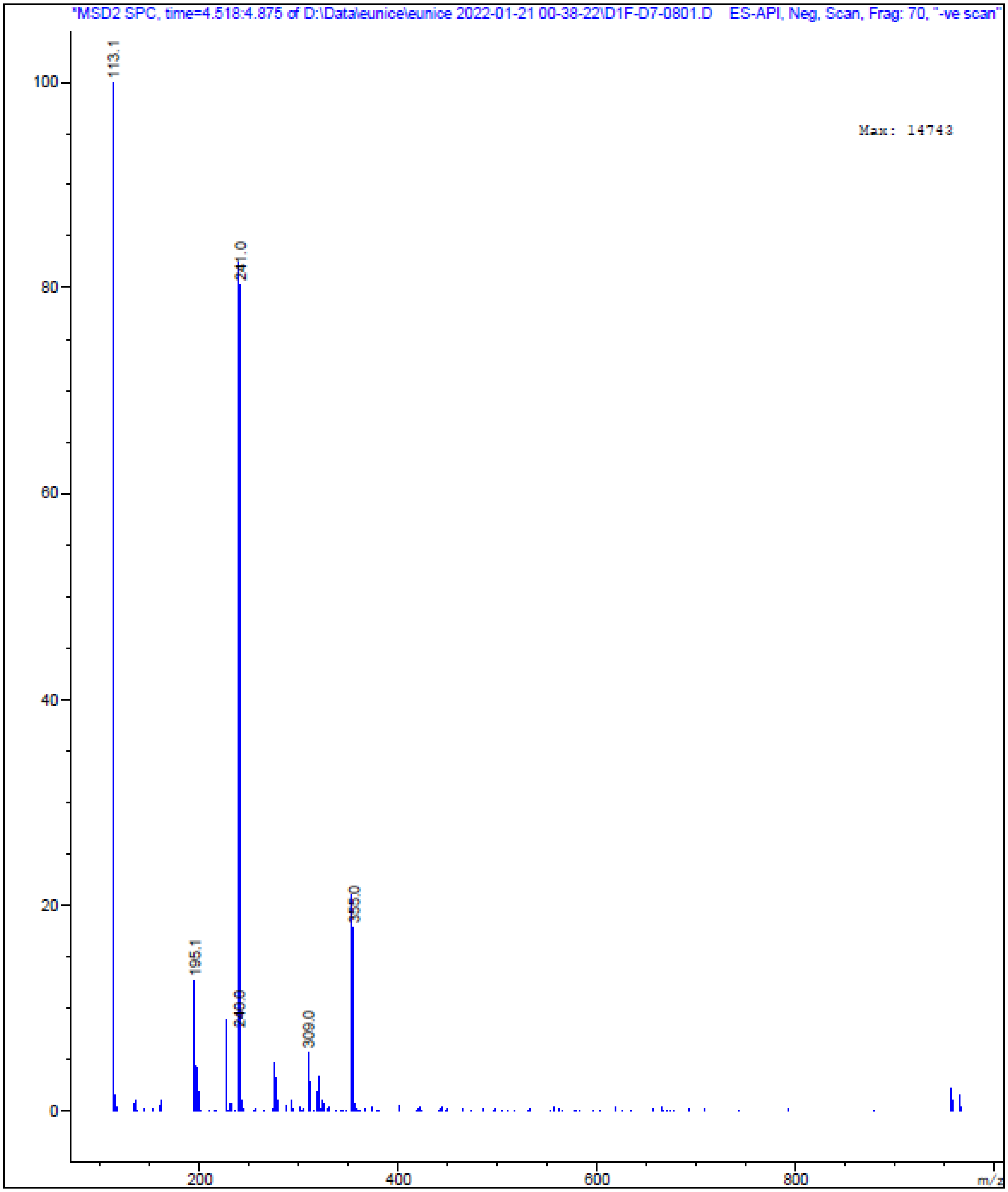

Supplementary Figure S9. HPLC spectra for bromination of 5-nitroindole (**1a**) with RebH 3-LSR Halogenase and reductase used for different experiments: (1) Partially purified 3-LSR, (2) Purified 3-LSR, (3) Purified 3-LSR and 5 µM RebF, (4) Purified 3-LSR and 50 µL fraction C7, (5) Purified 3-LSR and 5 µM purified AhpF and MS (ESI-) spectra of the currespnding brominated product . No di and tri substituted products were observed by mass extraction in MS.

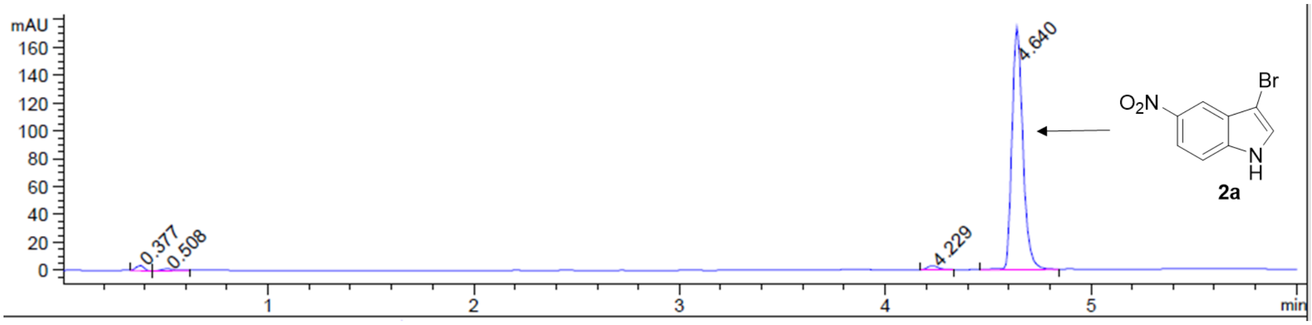

Supplementary Figure S10. HPLC spectrum of bromination of indole substrate **1a** produced by enzymatic halogenation using partially purified 3-LSR. No di and tri substituted product were observed by mass extraction in MS.

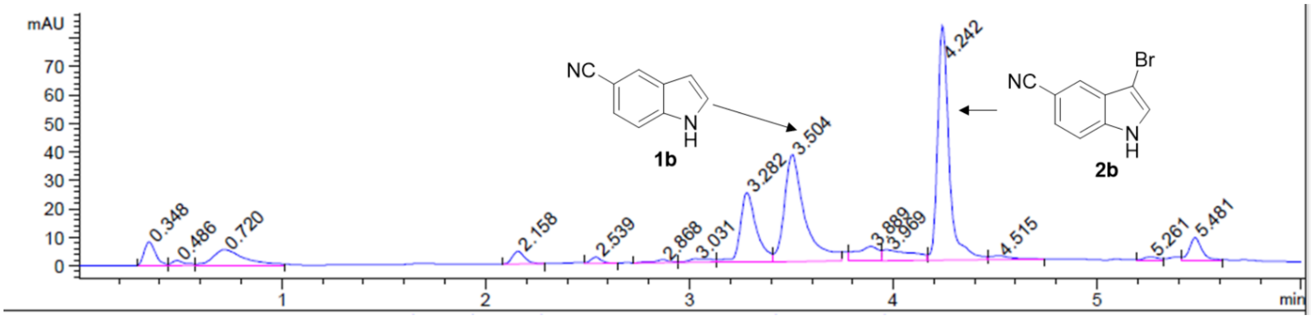

Supplementary Figure S11. HPLC spectrum of bromination of indole substrate **1b** produced by enzymatic halogenation using partially purified 3-LSR. No di and tri substituted product were observed by mass extraction in MS.

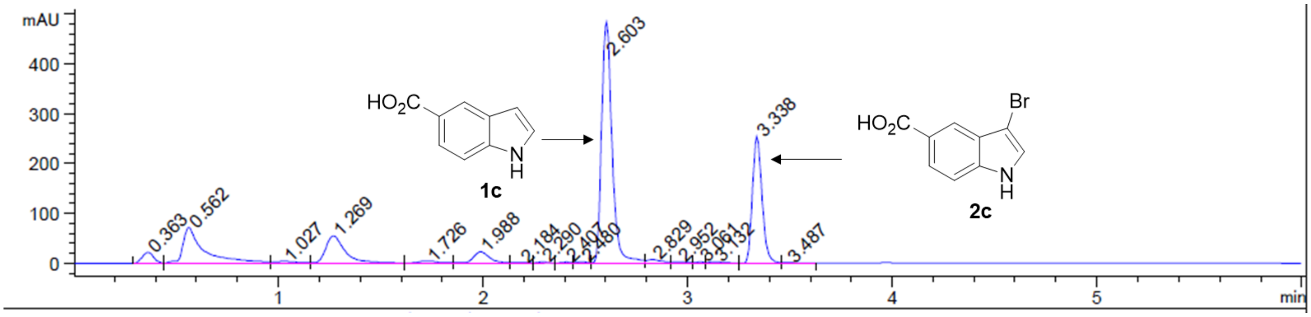

Supplementary Figure S12. HPLC spectrum of bromination of indole substrate **1c** produced by enzymatic halogenation using partially purified 3-LSR. No di and tri substituted product were observed by mass extraction in MS.

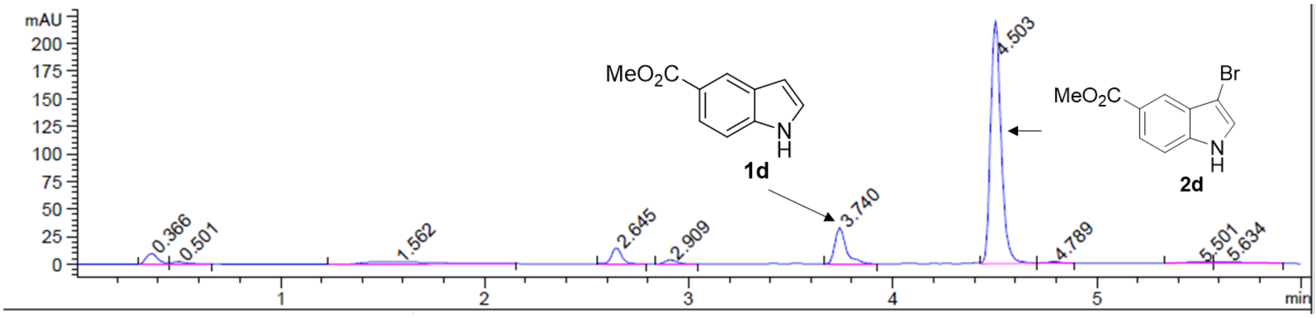

Supplementary Figure S13. HPLC spectrum of bromination of indole substrate **1d** produced by enzymatic halogenation using partially purified 3-LSR. No di and tri substituted product were observed by mass extraction in MS.

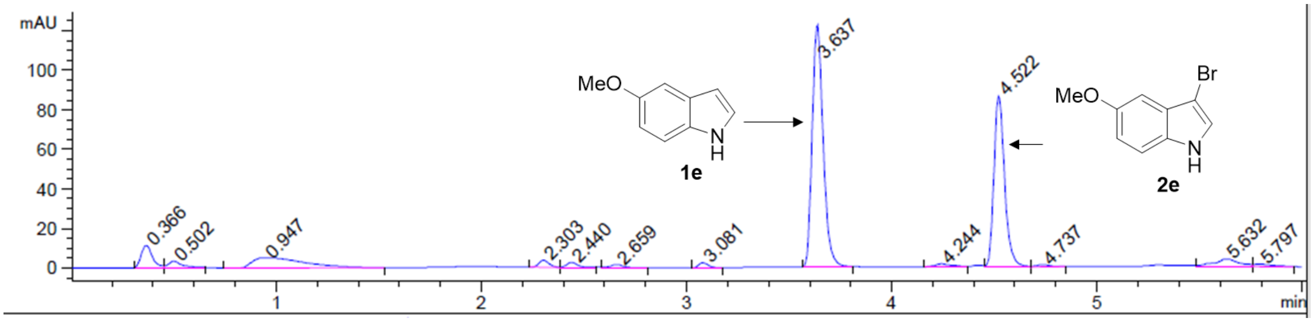

Supplementary Figure S14. HPLC spectrum of bromination of indole substrate **1e** produced by enzymatic halogenation using partially purified 3-LSR. No di and tri substituted product were observed by mass extraction in MS.

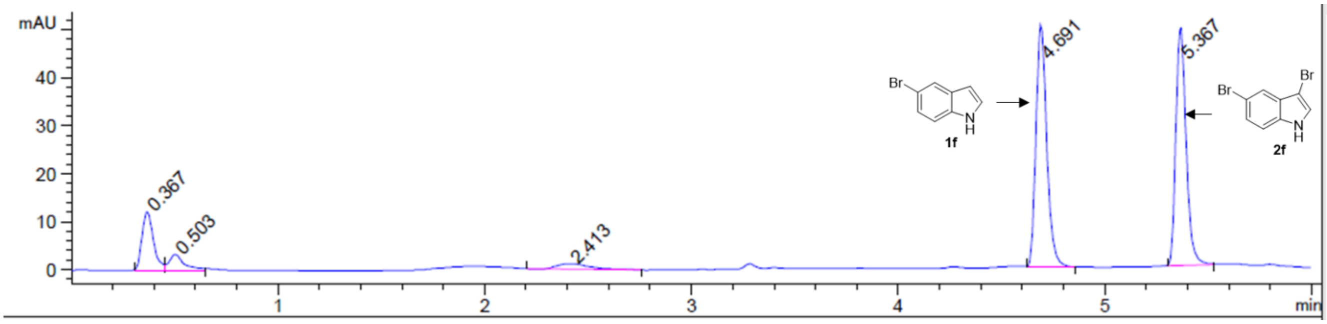

Supplementary Figure S15. HPLC spectrum of bromination of indole substrate **1f** produced by enzymatic halogenation using partially purified 3-LSR. No di and tri substituted product were observed by mass extraction in MS.

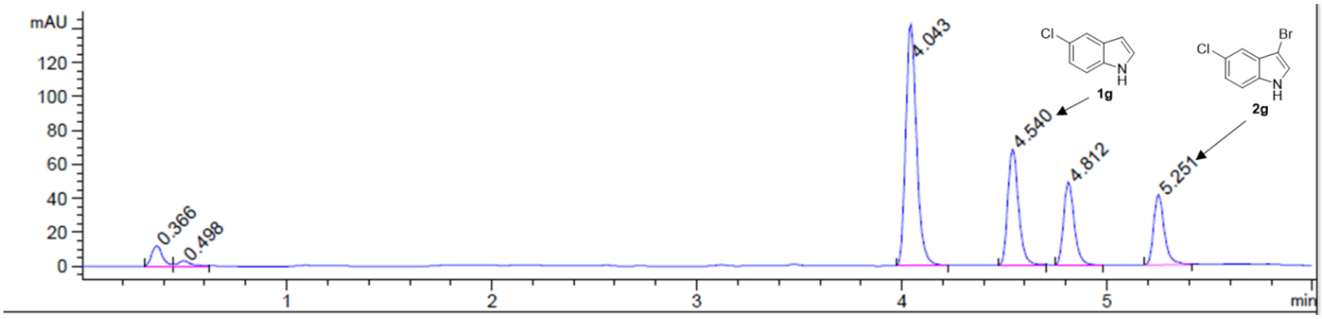

Supplementary Figure S16. HPLC spectrum of bromination of indole substrate **1g** produced by enzymatic halogenation using partially purified 3-LSR. No di and tri substituted product were observed by mass extraction in MS.

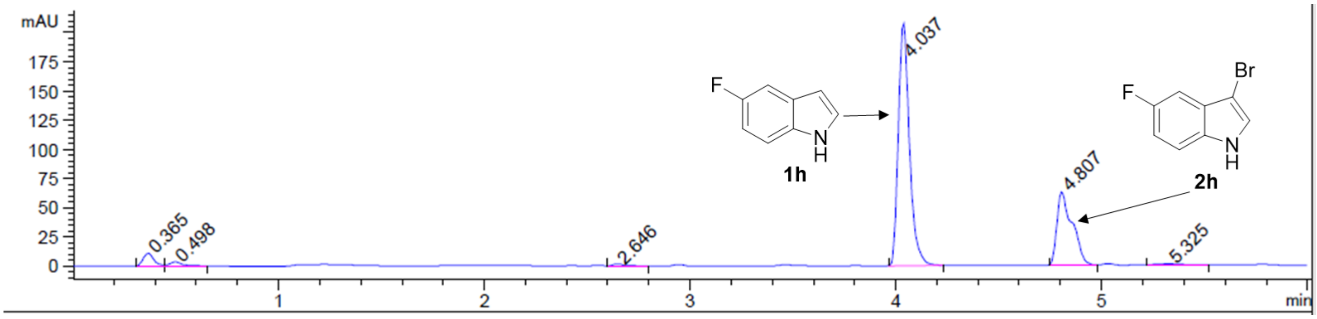

Supplementary Figure S17. HPLC spectrum of bromination of indole substrate **1h** produced by enzymatic halogenation using partially purified 3-LSR. No di and tri substituted product were observed by mass extraction in MS.

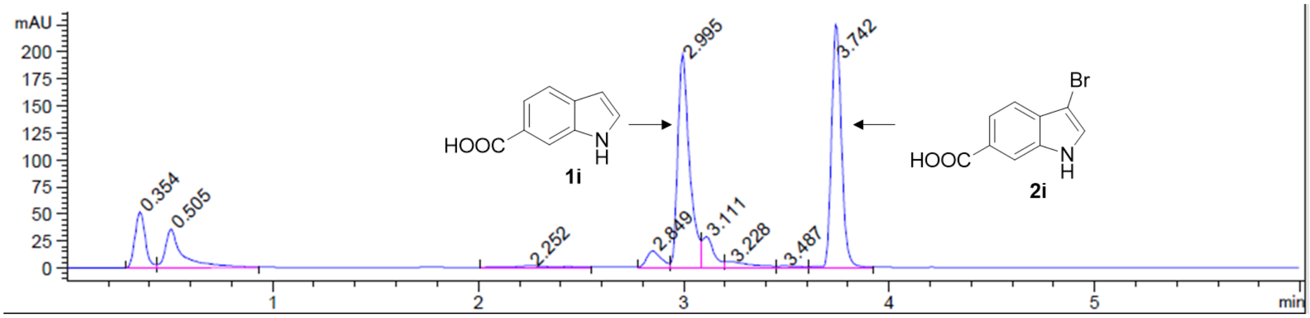

Supplementary Figure S18. HPLC spectrum of bromination of indole substrate **1i** produced by enzymatic halogenation using partially purified 3-LSR. No di and tri substituted product were observed by mass extraction in MS.

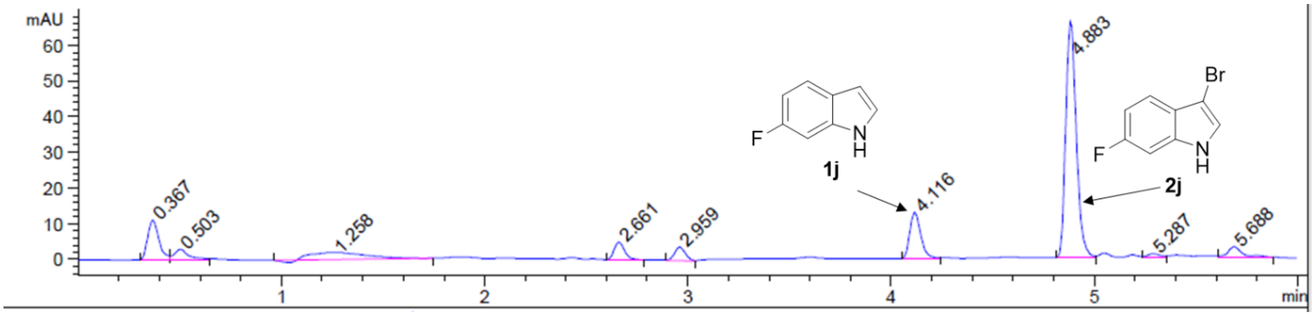

Supplementary Figure S19. HPLC spectrum of bromination of indole substrate **1j** produced by enzymatic halogenation using partially purified 3-LSR. No di and tri substituted product were observed by mass extraction in MS.

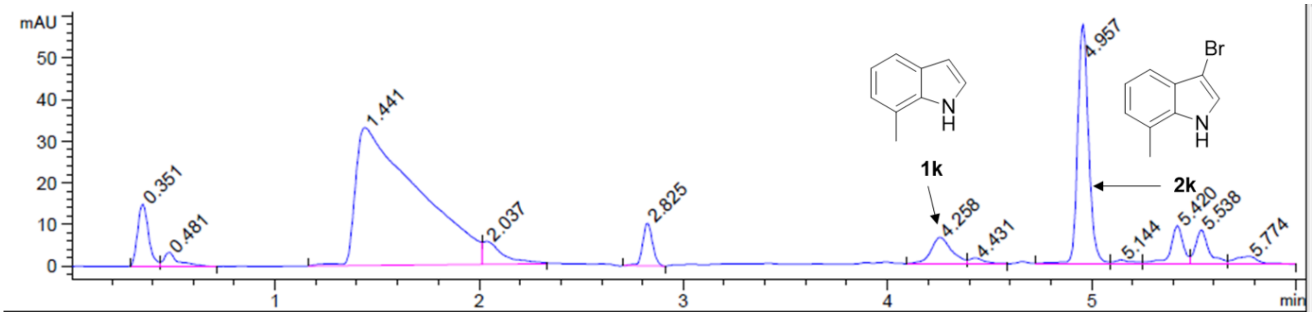

Supplementary Figure S20. HPLC spectrum of bromination of indole substrate **1k** produced by enzymatic halogenation using partially purified 3-LSR. No di and tri substituted product were observed by mass extraction in MS.

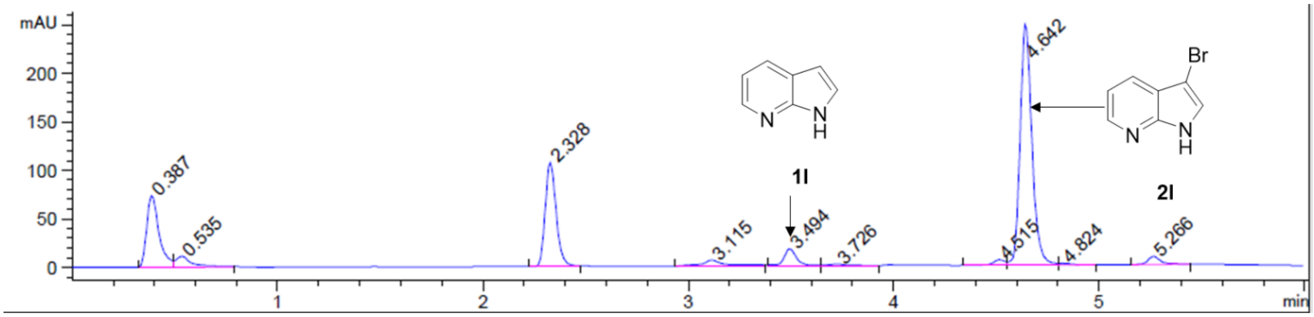

Supplementary Figure S21. HPLC spectrum of bromination of indole substrate **1l** produced by enzymatic halogenation using partially purified 3-LSR. No di and tri substituted product were observed by mass extraction in MS.

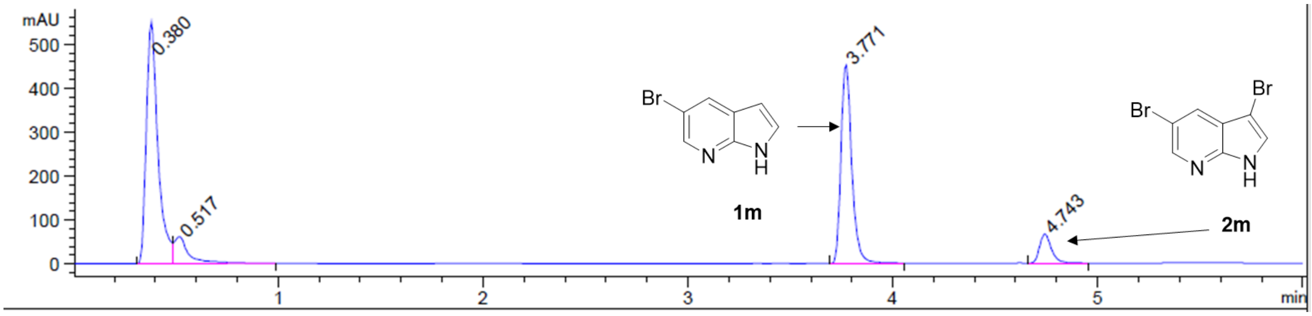

Supplementary Figure S22. HPLC spectrum of bromination of indole substrate **1m** produced by enzymatic halogenation using partially purified 3-LSR. No di and tri substituted product were observed by mass extraction in MS.

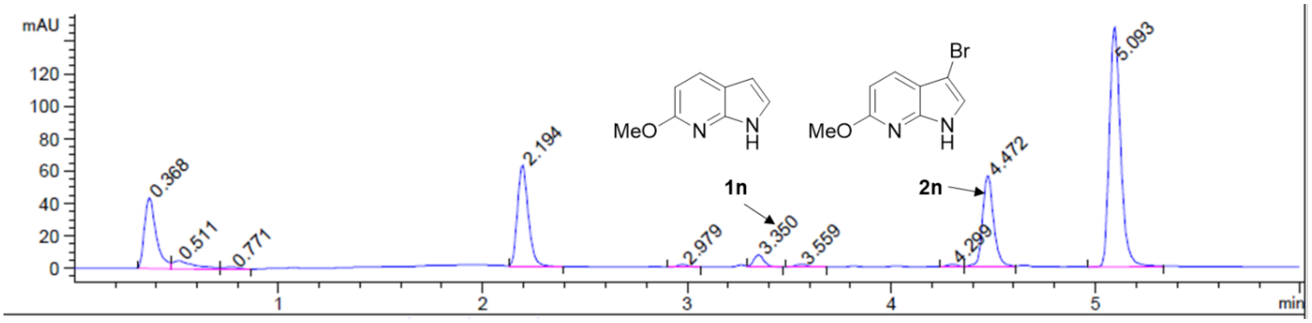

Supplementary Figure S23. HPLC spectrum of bromination of indole substrate **1n** produced by enzymatic halogenation using partially purified 3-LSR. No di and tri substituted product were observed by mass extraction in MS.

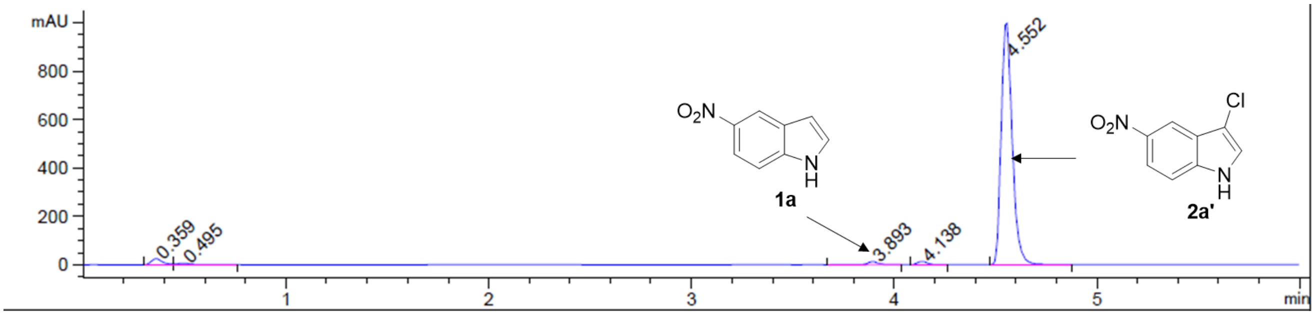

Supplementary Figure S24. HPLC spectrum of chlorination of indole substrate **1a** produced by enzymatic halogenation using partially purified 3-LSR. No di and tri substituted product were observed by mass extraction in MS.

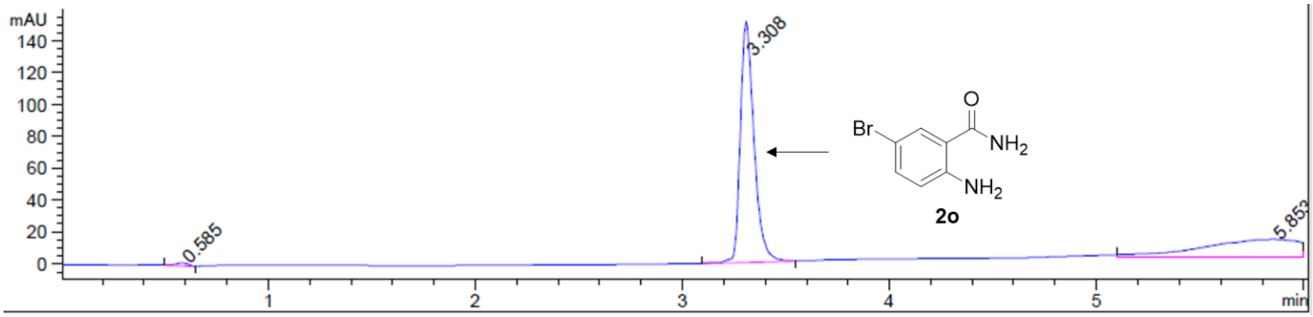

Supplementary Figure S25. HPLC spectrum of bromination of indole substrate **1o** produced by enzymatic halogenation using partially purified 3-LSR. No di and tri substituted product were observed by mass extraction in MS.

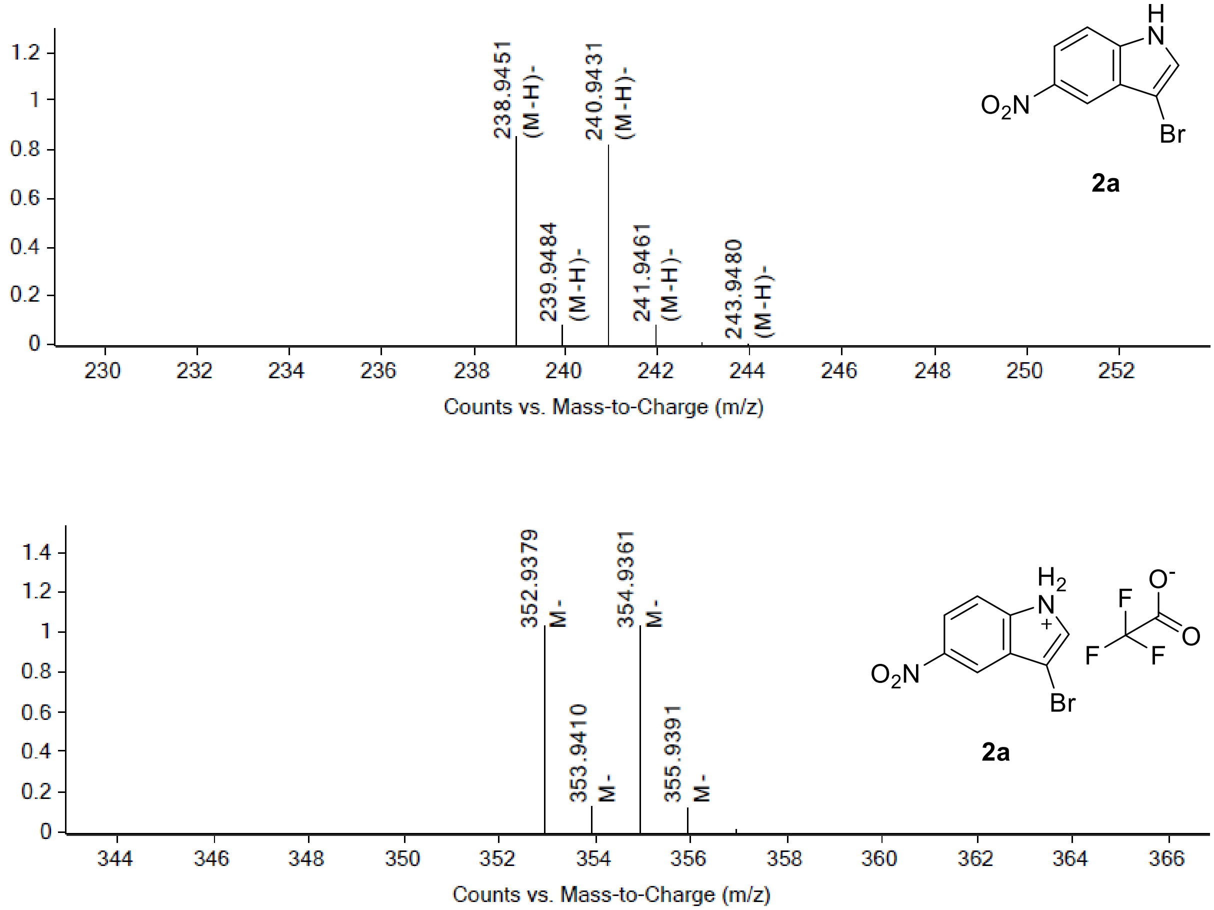

Supplementary Figure S26. HRMS spectrum of **2a** (top) and its triflate salt (bottom).

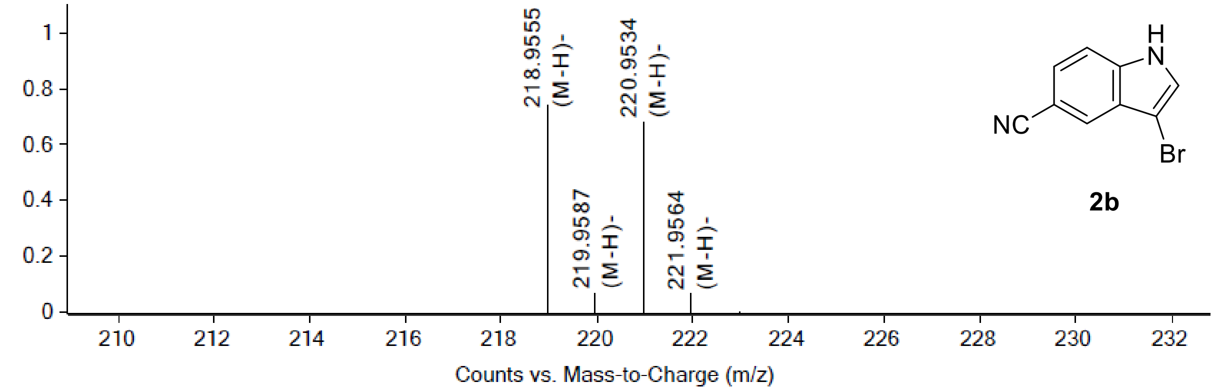

Supplementary Figure S27. HRMS spectrum of **2b**.

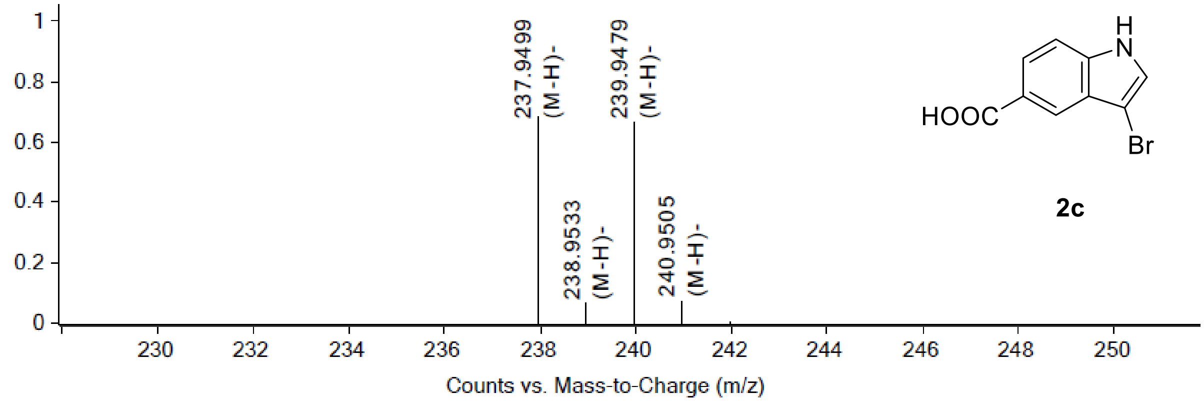

Supplementary Figure S28. HRMS spectrum of **2c**.

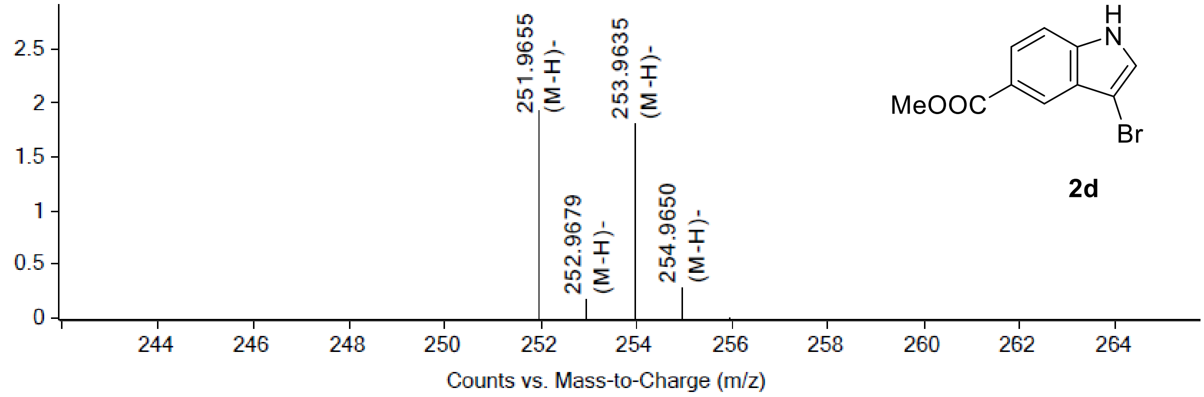

Supplementary Figure S29. HRMS spectrum of **2d**.

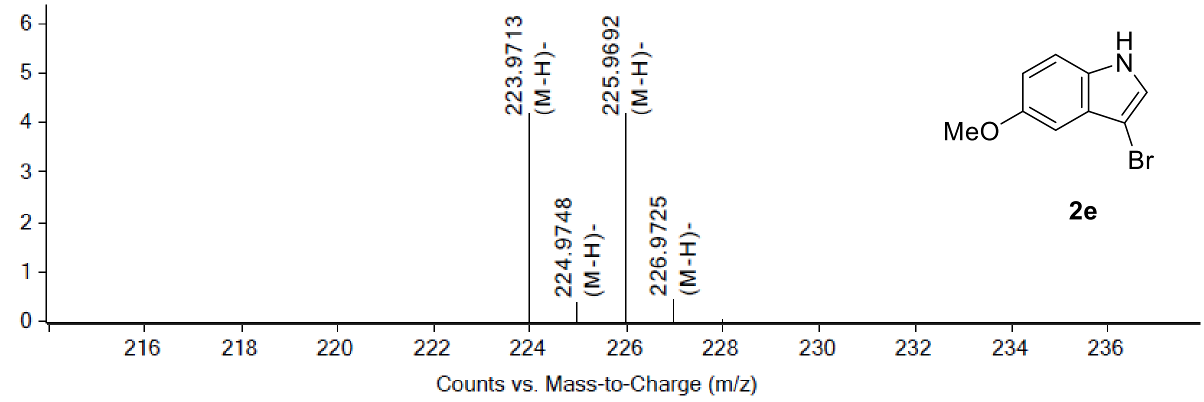

Supplementary Figure S30. HRMS spectrum of **2e**.

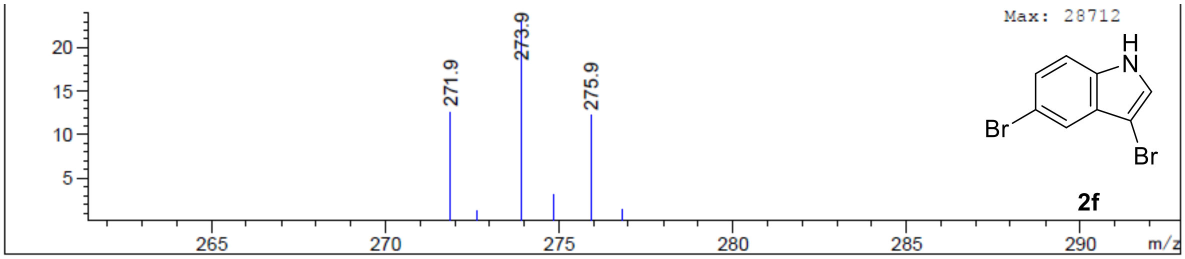

Supplementary Figure S31. LRMS spectrum of **2f**.

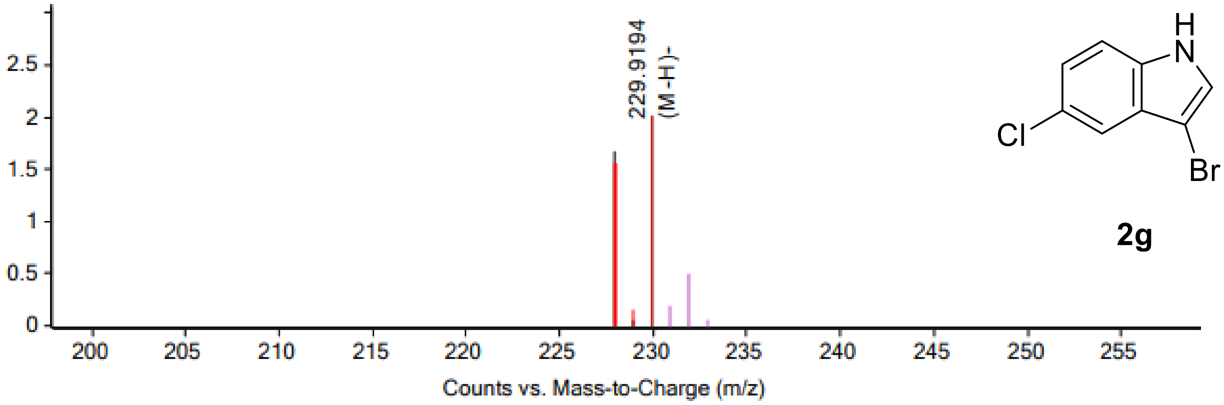

Supplementary Figure S32. HRMS spectrum of **2g**.

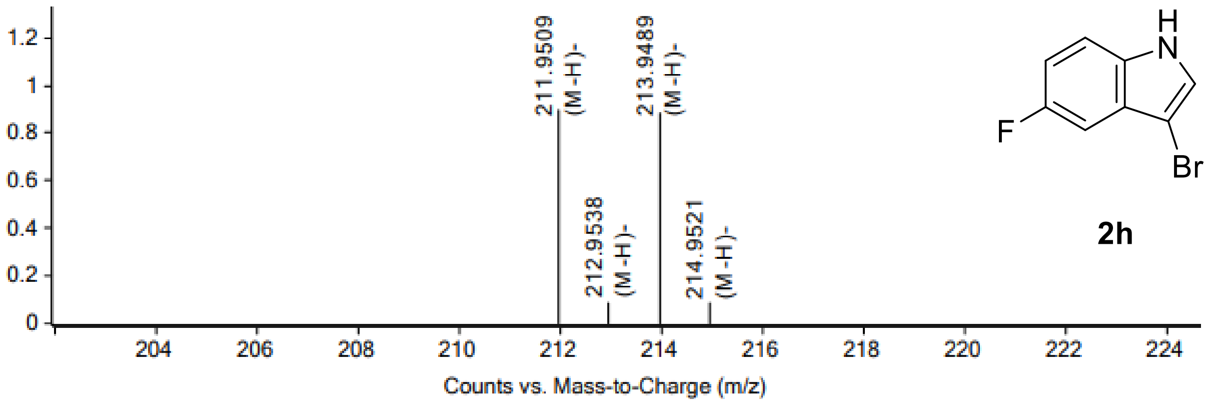

Supplementary Figure S33. HRMS spectrum of **2h**.

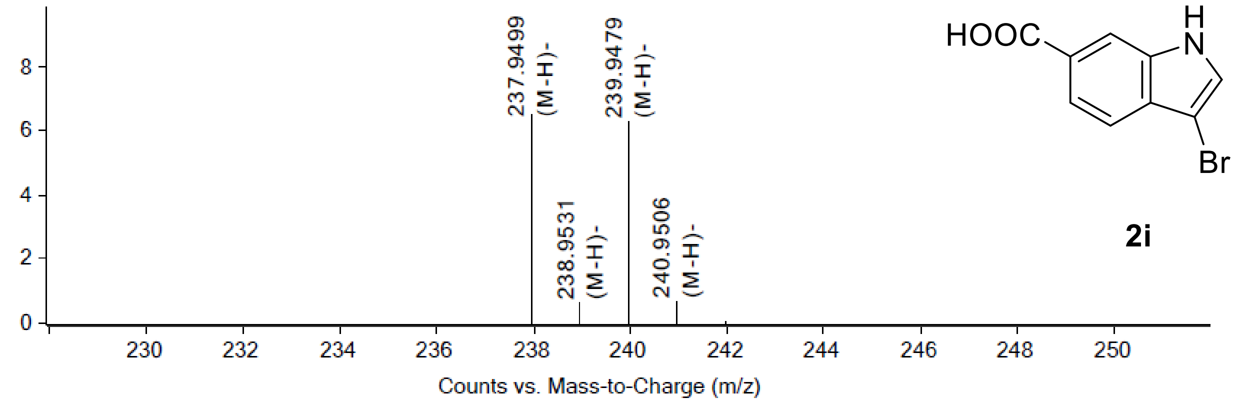

Supplementary Figure S34. HRMS spectrum of **2i**.

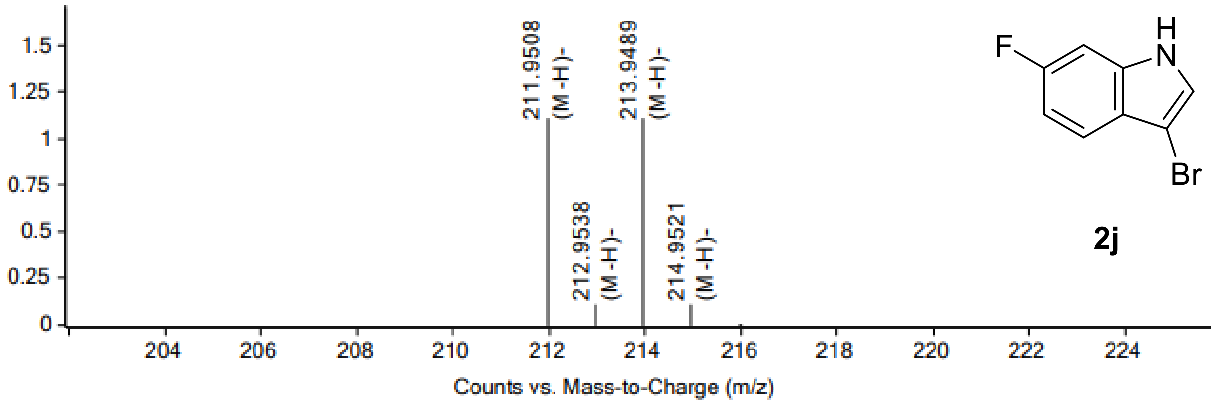

Supplementary Figure S35. HRMS spectrum of **2j**.

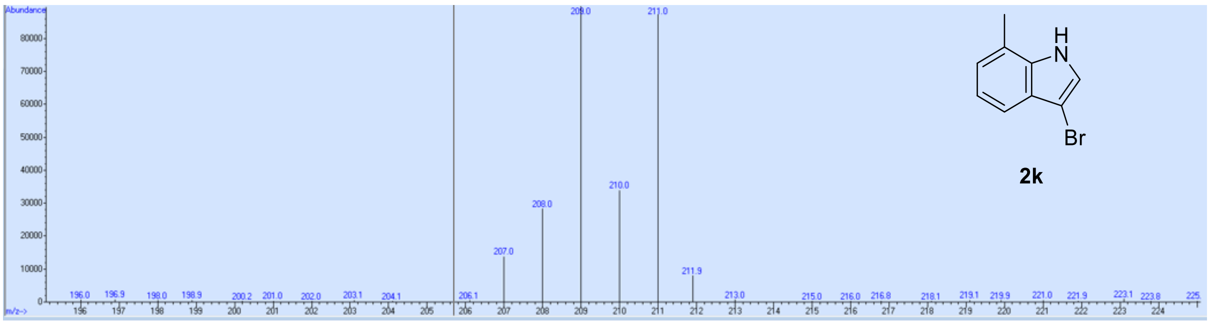

Supplementary Figure S36. LRMS spectrum of **2k**.

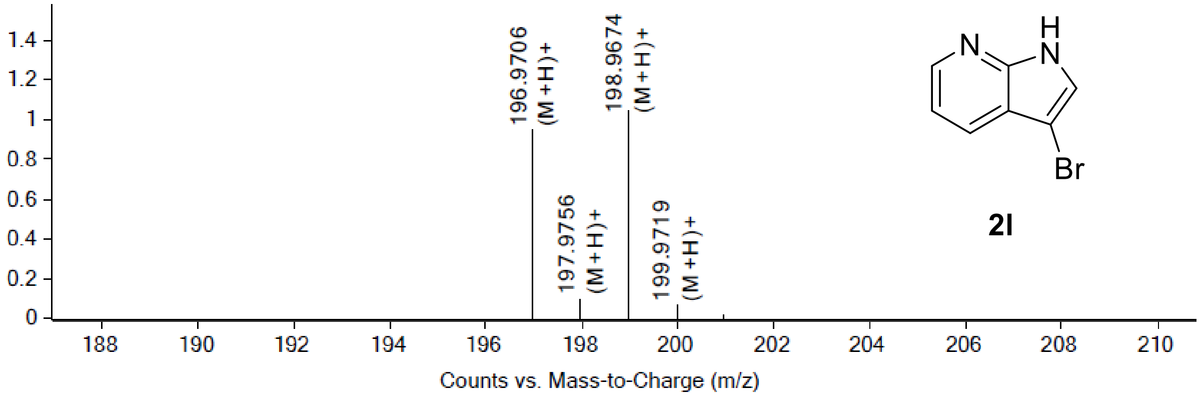

Supplementary Figure S37. HRMS spectrum of **2l**.

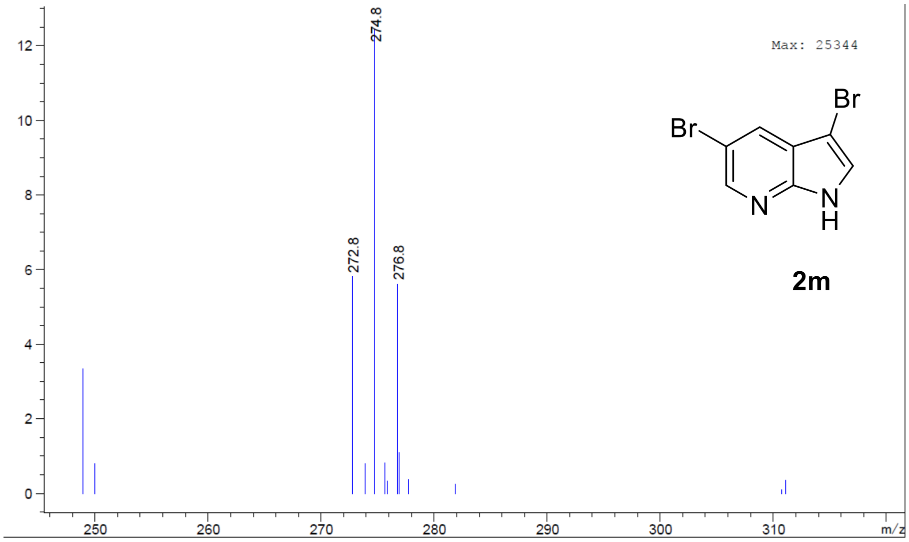

Supplementary Figure S38. LRMS spectrum of **2m**.

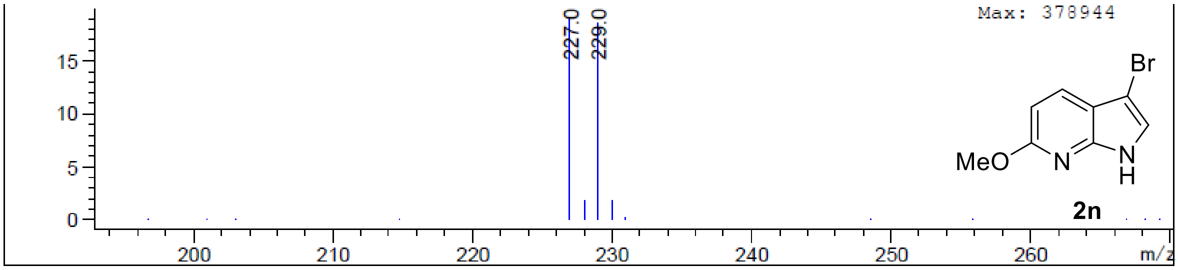

Supplementary Figure S39. LRMS spectrum of **2n**.

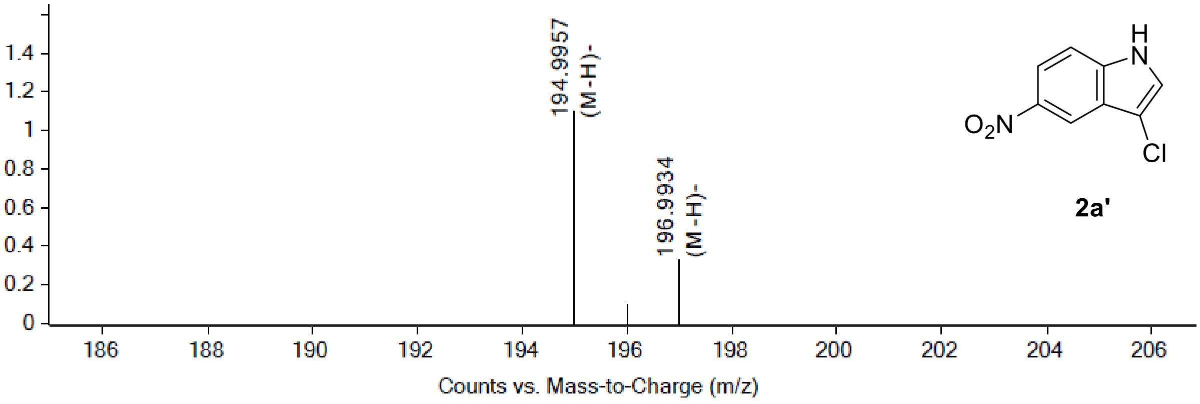

Supplementary Figure S40. HRMS spectrum of **2a'**.

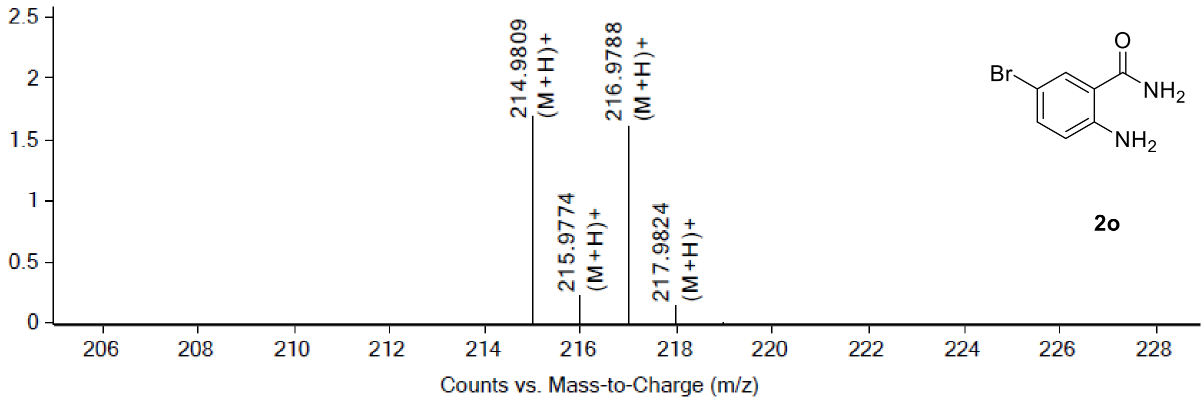

Supplementary Figure S41. HRMS spectrum of **2o**.

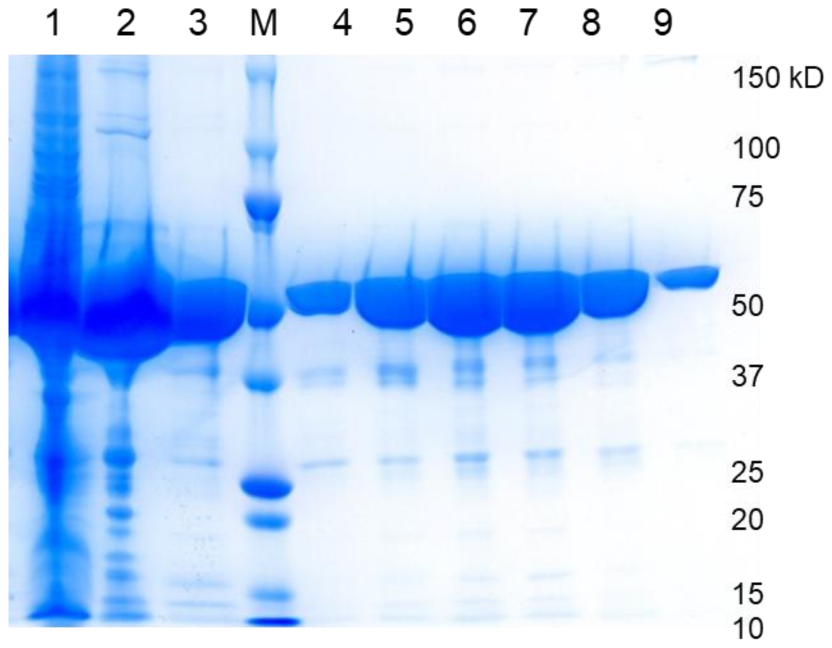

Supplementary Figure S42. Purification of recombinant AhpF protein. 1 = cell lysate supernatant, 2 and 3 = HisTrap elution with 100 mM and 500 mM imidazole respectively, 4 – 8 = anion-exchange fractions, 9 = size exclusion purified AhpF protein.

Supplementary Table S1. AhpF gene sequence for recombinant expression in *E. coli*

TTGGACACGAACATGAAAACGCAACTGAAAGCGTATCTGGAAAAGCTGACAAAGCCAGTGGAGC<br>TGATTGCTACCCTGGACGATTCGGCCAAATCTGCCGAAATCAAGGAGCTGCTGGCGGAGATCGC<br>AGAACTGAGTGACAAAGTGACTTTTAAGGAGGATAACAGCTTGCCTGTGCGTAAACCGAGTTTC<br>CTGATTACTAATCCGGGGAGTAATCAGGGTCCTCGTTTTGCGGGTAGTCCGTTAGGCCACGAAT<br>TTACATCGCTGGTATTAGCCCTGTTGTGGACTGGCGGGCACCCGTCTAAAGAAGCGCAATCACT

```
GCTGGAGCAGATTCGCCATATCGATGGGGATTTCGAATTCGAGACTTACTATAGTCTGTCTTGT
CACAATTGTCCGGATGTGGTACAGGCGCTGAATCTGATGTCAGTTCTGAATCCCCGCATTAAAC
ACACTGCCATCGACGGTGGTACGTTCCAAAATGAGATTACTGATCGTAATGTAATGGGGGTGCC
AGCGGTTTTTGTTAACGGCAAGGAATTCGGCCAGGGGCGTATGACGTTAACGGAAATCGTAGCC
AAAATCGATACCGGTGCCGAGAAGCGTGCGGCCGAGGAACTGAATAAACGTGATGCGTATGATG
TACTGATTGTTGGGTCCGGTCCAGCAGGTGCCGCCGCCGCTATTTACTCAGCACGTAAAGGTAT
CCGTACCGGGTTGATGGGTGAGCGTTTTGGTGGTCAAATTCTGGACACTGTCGATATTGAAAAC
TACATCTCTGTACCAAAGACTGAAGGGCAGAAACTTGCCGGTGCCTTGAAAGTTCATGTCGACG
AGTATGACGTCGATGTAATTGACTCCCAAAGTGCCTCGAAACTGATTCCCGCTGCTGTTGAGGG
CGGCCTGCACCAAATCGAGACCGCATCCGGTGCAGTACTGAAGGCGCGCTCGATCATTGTTGCT
ACTGGTGCAAAATGGCGCAACATGAACGTGCCTGGGGAAGACCAGTACCGCACGAAAGGTGTTA
CTTATTGTCCTCATTGCGATGGTCCACTGTTCAAAGGTAAACGTGTGGCTGTTATCGGTGGCGG
TAACTCGGGCGTCGAGGCTGCAATCGATCTGGCAGGTATTGTCGAGCACGTAACATTACTGGAG
TTTGCACCTGAAATGAAGGCCGACCAAGTACTGCAGGATAAGTTGCGCTCCCTGAAAAATGTCG
ACATCATCCTGAACGCGCAGACCACGGAGGTGAAAGGTGATGGTTCTAAGGTGGTTGGTCTGGA
ATATCGCGATCGTGTCTCAGGTGACATTCATAACATTGAACTTGCCGGTATTTTTGTGCAAATT
GGCCTGCTGCCAAATACAAACTGGTTAGAGGGTGCCGTCGAACGCAACCGCATGGGGGAAATCA
TTATTGATGCCAAATGTGAAACCAATGTAAAAGGTGTATTTGCAGCCGGTGACTGTACGACTGT
GCCGTATAAGCAGATTATTATCGCGACAGGTGAAGGGGCAAAGGCATCACTGAGCGCTTTTGAC
TATCTGATCCGTACGAAGACAGCT
```

Reference:

1. Hugon, B.; Anizon, F.; Bailly, C.; Golsteyn, R. M.; Pierré, A.; Léonce, S.; Hickman, J.; Pfeiffer, B.; Prudhomme, M., Synthesis and biological activities of isogranulatimide analogues. *Bioorganic & Medicinal Chemistry* **2007,** *15* (17), 5965-5980.

2. Ismail, M.; Frese, M.; Patschkowski, T.; Ortseifen, V.; Niehaus, K.; Sewald, N., Flavin-Dependent Halogenases from Xanthomonas campestris pv. campestris B100 Prefer Bromination over Chlorination. *Advanced Synthesis & Catalysis* **2019,** *361* (11), 2475-2486.

3. Cooper, S. P.; Booker-Milburn, K. I., A Palladium(II)-Catalyzed C□H Activation Cascade Sequence for Polyheterocycle Formation. *Angewandte Chemie International Edition* **2015,** *54* (22), 6496-6500.

4. Manzoni, L.; Zucal, C.; Maio, D. D.; D'Agostino, V. G.; Thongon, N.; Bonomo, I.; Lal, P.; Miceli, M.; Baj, V.; Brambilla, M.; Cerofolini, L.; Elezgarai, S.; Biasini, E.; Luchinat, C.; Novellino, E.; Fragai, M.; Marinelli, L.; Provenzani, A.; Seneci, P., Interfering with HuR–RNA Interaction: Design, Synthesis and Biological Characterization of Tanshinone Mimics as Novel, Effective HuR Inhibitors. *Journal of Medicinal Chemistry* **2018,** *61* (4), 1483-1498.

5. Yan, J.; Ni, T.; Yan, F., Simple and efficient procedures for selective preparation of 3-haloindoles and 2,3-dihaloindoles by using 1,3-dibromo-5,5-dimethylhydantoin and 1,3-dichloro-5,5-dimethylhydantoin. *Tetrahedron Letters* **2015,** *56* (9), 1096-1098.

6. Guney, T.; Lee, J. J.; Kraus, G. A., First Inverse Electron-Demand Diels–Alder Methodology of 3-Chloroindoles and Methyl Coumalate to Carbazoles. *Organic Letters* **2014,** *16* (4), 1124-1127.

7. Wang, Y.-W.; Zheng, L.; Jia, F.-C.; Chen, Y.-F.; Wu, A.-X., Oxidative ring-opening of isatins for the synthesis of 2-aminobenzamides and 2-aminobenzoates. *Tetrahedron* **2019,** *75* (11), 1497-1503.
